# Uncovering the Dynamic CO_2_ Gas Uptake Behavior of CALF‐20 (Zn) under Varying Conditions via Positronium Lifetime Analysis

**DOI:** 10.1002/smll.202500544

**Published:** 2025-02-25

**Authors:** Ahmed G. Attallah, Volodymyr Bon, Eric Hirschmann, Maik Butterling, Andreas Wagner, Radosław Zaleski, Stefan Kaskel

**Affiliations:** ^1^ Institute of Radiation Physics Helmholtz‐Zentrum Dresden – Rossendorf 01328 Dresden Germany; ^2^ Physics Department Faculty of Science Minia University Minia 61519 Egypt; ^3^ Chair of Inorganic Chemistry I Technische Universität Dresden D‐01062 Dresden Germany; ^4^ Institute of Physics Maria Curie‐Sklodowska University Lublin 20‐031 Poland; ^5^ Present address: Delft University of Technology, Faculty of Applied Sciences Department of Radiation Science & Technology Mekelweg 15 JB, Delft NL‐2629 Netherlands

**Keywords:** CALF‐20, CO_2_ capture and storage, environmental gas uptake, positron annihilation

## Abstract

Carbon dioxide (CO_2_) is a major greenhouse gas contributing to global warming. Adsorption in porous sorbents offers a promising method for CO_2_ capture and storage. The zinc‐triazole‐oxalate‐based Calgary framework 20 (CALF‐20) demonstrates high CO_2_ capacity, low H_2_O affinity, and low adsorption heat, enabling energy‐efficient and stable performance over multiple cycles. This study examines CO_2_ adsorption mechanism in CALF‐20 using positron annihilation lifetime spectroscopy (PALS), in situ powder X‐ray diffraction (PXRD), and gas adsorption experiments under varying temperatures and humidity levels. Variable‐temperature PALS experiments demonstrate that CO₂ molecules are spatially localized within the CALF‐20 cages, leaving temperature‐ and pressure‐dependent gaps. CO_2_ begins at cage centers, forming 1D chains, and ultimately adheres to pore walls. Interestingly, positronium intensity correlates with the Langmuir‐Freundlich isotherm, reflecting gas uptake behavior. Moreover, under pure relative humidity (RH), water molecules form isolated clusters or small oligomers at low RH, transitioning to hydrogen‐bonded networks above 35 %RH, significantly altering free volumes. In humid CO₂ conditions, competitive interactions arise: CO₂ initially disrupts water propagation, but higher RH leads to extensive water networks filling the framework. The synergy between in situ‐PALS, in situ‐PXRD, and gas adsorption techniques provides comprehensive insights into CALF‐20′s potential for efficient CO_2_ capture under varying conditions.

## Graphical abstract

CALF‐20 is stable between 253 K and 373 K. CO_2_ adsorption on CALF‐20 leaves gaps and shows higher CO_2_ uptake at T < RT and positron annihilation lifetime spectroscopy (PALS) reproduces the sorption isotherm. CO_2_ molecules are shifting from the cage centers at high adsorption pressure. Water‐loaded CALF‐20 requires minimal energy for purging. CO_2_ remains selective over H_2_O at low humidity, whereas at high humidity H‐bonded H_2_O dominates.

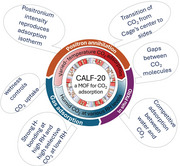

## 1 Introduction

During the world climate conference in 2021, 153 countries agreed on a Glasgow Climate Pact keeping the 1.5 °C target of global warming temperature within reach, with plans to revisit and tighten their emissions targets by 2030.^[^ ^1^ ^]^ The persistent rise in human‐driven CO_2_ emissions continues to intensify climate change, underscoring the critical need for effective carbon capture strategies.^[^ ^2^ ^]^ As temperatures rise and environmental challenges unfold, the demand for innovative approaches to reduce carbon emissions grows steadily. Separation and capture of greenhouse gases from dilute emissions are among the seven most important separation processes, which are responsible for 45–55% of energy consumption in industry.^[^ ^3^ ^]^ Various approaches, such as amine scrubbing, membrane separation, cryogenic distillation, and chemical looping,^[^ ^4^ ^]^ have been explored to address this challenge, and the adsorption of CO_2_ from air under different environmental conditions emerges as a promising pathway due to its cost‐effectiveness and operational simplicity.^[^ ^5^, ^6^ ^]^

It is desirable to choose adsorbents that have a high CO_2_ capacity, a low N_2_ and H_2_O affinity, and a relatively low CO_2_ adsorption heat to conserve energy while retaining good CO_2_ purity, recovery, and productivity.^[^ ^7^, ^8^ ^]^ Various types of adsorbents are being investigated for CO_2_ capture, such as activated carbon,^[^ ^9^ ^]^ zeolites,^[^ ^10^ ^]^ silica gel,^[^ ^11^ ^]^ metal oxides,^[^ ^12^ ^]^ and metal‐organic frameworks (MOFs). The main challenges of well‐established industrial adsorbents such as zeolites and silica gels are quite high adsorption enthalpy and co‐adsorption of water, which is in most cases present in the gas mixture. On the other hand, activated carbons tend to exhibit non‐polar surface and therefore low selectivity towards CO_2_.

MOFs are a class of hybrid nanoporous crystalline materials that are constructed of metal ions or clusters connected by organic linkers using modular building principles.^[^ ^13^ ^]^ MOFs have attracted much attention as potential adsorbents for CO_2_ capture due to their high surface area, tunable pore size and shape, adjustable functionality, and structural diversity.^[^ ^14^, ^15^, ^16^ ^]^ Different approaches are proposed for enhancing CO_2_ capture in MOFs including the introduction of coordinative unsaturated metal sites, amine functionalization, modulation of hydrophobicity of the pores, and the mixture thereof.^[^ ^14^, ^16^, ^17^, ^18^ ^]^ At the forefront of this strive is Calgary Framework 20 (CALF‐20) (Zn), a zinc‐based MOF, first reported by Shimizu et al.,^[^ ^19^ ^]^ distinguished by its hydrophobicity, high scalability, and robust structural integrity. With a surface area of ca. 530 m^2^ g^−1[^ ^19^, ^20^ ^]^ and an average pore size of 6–7 Å,^[^ ^20^, ^21^, ^22^ ^]^ CALF‐20 (Zn) demonstrates a remarkable capacity and selective CO_2_ adsorption^[^ ^23^ ^]^ at desired conditions.^[^ ^21^ ^]^ Its unique framework, composed of 1,2,4‐triazole and oxalate ligands confers stability in harsh environments.^[^ ^21^ ^]^ The necessity of capturing CO_2_ under fluctuating conditions is emphasized by the varying concentrations of CO_2_ found in diverse industrial emissions and atmospheric contexts. CALF‐20 (Zn) exhibits exceptional versatility in maintaining operational efficacy across a range of temperatures and pressures, positioning it as an indispensable tool in global efforts to mitigate CO_2_ emissions.^[^ ^5^ ^]^ Its capabilities are boosted through the incorporation of functional groups that amplify its affinity to CO_2_, thereby optimizing the adsorption process.^[^ ^2^ ^]^ Within the PrISMa (process‐informed design of tailor‐made sorbent materials) platform, which includes the comprehensive screening of materials including CO_2_ source, destination, capture technology, and geographical regions, CALF‐20 was identified among the top‐performing materials for further study in terms of more detailed process modelling and investigation for technological development.^[^ ^24^ ^]^ Besides that, recently the guest‐induced flexibility in CALF‐20 was discovered.^[^ ^20^, ^25^ ^]^ It was found that under humid conditions, the structure undergoes a phase transition from α‐CALF‐20 to β‐CALF‐20, accompanied by a minor contraction and adsorption of water molecules.^[^ ^20^ ^]^ This transition was studied by a combination of in situ PXRD and total scattering. However, the authors pointed out the challenges associated with characterizing CO_2_ adsorption properties of β‐CALF‐20 phase due to the reversibility and fast kinetics of α‐β‐α transitions. Further in‐depth structural studies result in a number of phases, most of which are close to β‐phase in terms of porosity.^[^ ^25^ ^]^

To this end, studying gas adsorption dynamics on the highly scalable CALF‐20 MOFs is essential to gain insights into the complex processes at play. This is important for CO_2_ capture under varying conditions, where the adsorption behavior may shift. This approach enables the elucidation of the dynamic interactions between gas molecules and the porous structure of CALF‐20, potentially offering a clearer understanding of CALF‐20′s performance in different environmental conditions.^[^ ^26^ ^]^

For a comprehensive insight into the CO_2_ adsorption dynamics of CALF‐20 (Zn), positron annihilation lifetime spectroscopy (PALS) stands out as an invaluable tool.^[^ ^27^, ^28^, ^29^, ^30^ ^]^ PALS can differentiate the subtleties of pore filling and emptying processes,^[^ ^28^, ^29^, ^31^ ^]^ providing insights into the adsorption mechanisms and molecular packing within the porous materials, e.g., MOFs. This non‐destructive technique has proven to be sensitive to conformational, structural, and microenvironmental transformations, which are crucial in understanding the molecular organization and transport properties within CALF‐20 (Zn). More details about porosimetry by PALS are available in Appx. S1 (Supporting Information). Briefly, PALS utilizes the annihilation characteristics—quantified by lifetime and intensity—of ortho‐positronium (o‐Ps), a quasi‐stable bound state formed by an electron and a positron with parallel spins (Appx. S1, Supporting Information), to probe the microstructural properties of materials.^[^ ^28^, ^32^, ^33^, ^34^, ^35^, ^36^ ^]^ In porous materials, o‐Ps annihilates with a lifetime determined by probability of its bound positron annihilation with an electron of parallel spin from the pore walls (in empty pores) or from adsorbents and guests within the pores. This process, known as “pick‐off” annihilation, occurs at a rate that depends on the size of the pores and the accommodated substances. Consequently, the lifetime of o‐Ps is exceptionally sensitive to the local electronic environment (due to adsorption, for example) and can provide detailed information about changes in size and shape of unoccupied spaces. Meanwhile, o‐Ps intensity indicates the formation and trapping probabilities of o‐Ps, which are influenced by pore density, connectivity, surface area, and chemistry.^[^ ^37^, ^38^, ^39^, ^40^ ^]^ In this regard, PALS can be employed as a complementary tool alongside other chemical and spectroscopic methods to characterize CO_2_ adsorption in CALF‐20 under environmental conditions, particularly in scenarios where some of these methods face limitations related to temperature, pressure, or humidity. Additionally, the processes of adsorption and pore filling can result in the formation of closed or restricted pores that traditional gas adsorption methods may fail to detect, whereas o‐Ps can effectively identify these structures. Therefore, we believe that PALS is an informative new addition for understanding the adsorption dynamics at different conditions of CALF‐20, which can lead to improvements in the material's design and functionality, making it more effective in reducing CO_2_ emissions.

To advance our understanding of CO_2_ capture, this study investigates the adsorption dynamics on CALF‐20 (Zn) across various conditions taking advantage from PALS capabilities. This entails conducting precise treatments including a control of the sample temperature between 253 K and 373 K under vacuum, followed by in situ CO_2_ adsorption experiments and full‐pressure scans within the same temperature range during PALS measurements. Furthermore, we executed comprehensive full‐scale humid CO_2_ adsorption experiments, spanning humidity levels from 11% to 98%. Additionally, our investigation is complemented by in situ‐PXRD (powder X‐ray diffraction) analysis and physisorption experiments, conducted under identical conditions allowing us to compare the results with in situ gas adsorption data obtained via PALS, thus offering a holistic understanding of CALF‐20′s adsorption behavior.

## 2 Results and Discussions

Prior to any experimental procedures, the as‐received sample was annealed at 423 K for 10 h under a dynamic vacuum of ca. 1.5 × 10^−6^ mbar, as detailed in the method section.

### 2.1 Thermal Effect on CALF‐20 Porosity

One primary objective of this study was to investigate CO_2_ adsorption dynamics in CALF‐20 under different temperatures. In order to distinguish temperature effects from adsorption induced deformation, we initially conducted PALS measurements^[^ ^41^ ^]^ at varying temperature over a range from 253 to 373 K in a vacuum (1.5 × 10^−6^ mbar), with 2 h intervals per step. The activated sample was first heated to 373 K, then gradually cooled to 253 K at a heating/cooling rate of 2.5 K min^−1^.

PALS analysis using PALSFit^[^ ^42^ ^]^ revealed that there are two resolvable o‐Ps components with distinct lifetimes (ca. 4.5‐4.34 ns and 33.3‐34.5 ns, **Table** **1**) in CALF‐20. The short‐lived o‐Ps lifetime (τ_cage_) corresponds to square cross‐section channels of about 0.60‐0.64 nm, closely matching the crystallographically calculated cages of CALF‐20 in the α‐phase (Appx. S2 and Figure S1, Supporting Information). The long‐lived o‐Ps lifetime (τ_grain_) corresponding to gaps of around 1.5 nm is commonly interpreted as annihilation between grains (shown in Figure S2, Supporting Information) as noted in Table 1. This component may also include a fraction of o‐Ps annihilating from delocalized Bloch states as discussed below. The analysis indicates also that about one‐quarter (I_cage_ + I_grain_ in Table 1) of positrons in CALF‐20 form o‐Ps. The results in Table 1 suggest a weak dependence of o‐Ps parameters, and thus cages in CALF‐20 remain in the α‐phase over the 253 to 373 K temperature range in a vacuum. The cage size, as probed by o‐Ps in Table 1, negligibly increases with increasing the temperature between 253 and 373 K. Therefore, any greater changes observed during CO_2_ dosing in the next section are likely attributable to the adsorption process.

**Table 1:** Variations of o‐Ps parameters and the corresponding pore sizes in CALF‐20 at different temperatures under vacuum.

T [K]	τ_cage_ [ns]	τ_grain_ [ns]	d_cage_ [nm]	d_grain_ [nm]	I_cage_ [%]	I_grain_ [%]
253	4.34 ± 0.03	33.75 ± 0.47	0.60 ± 0.002	1.53 ± 0.01	13.11 ± 0.06	9.91 ± 0.05
273	4.47 ± 0.04	34.53 ± 0.49	0.61 ± 0.002	1.55 ± 0.01	12.81 ± 0.06	10.26 ± 0.05
295	4.54 ± 0.04	33.92 ± 0.47	0.61 ± 0.003	1.54 ± 0.01	12.49 ± 0.06	10.54 ± 0.05
320	4.75 ± 0.04	34.15 ± 0.47	0.63 ± 0.003	1.55 ± 0.01	12.11 ± 0.06	11.00 ± 0.05
350	4.72 ± 0.05	33.28 ± 0.44	0.62 ± 0.003	1.53 ± 0.01	11.83 ± 0.06	11.40 ± 0.05
373	4.95 ± 0.05	33.45 ± 0.46	0.64 ± 0.003	1.53 ± 0.01	11.40 ± 0.06	11.64 ± 0.06

### 2.2 Isothermal CO_2_ Adsorption at Varying Temperatures

To explore the CO_2_ adsorption course in CALF‐20, we investigated its temperature‐dependent adsorption using PALS. In the following, we have discussed the lifetimes and intensities separately. The discussion of hysteresis noticed in some curves and variation of τ_grain_ are detailed in Appx. S3 and Figure S3 (Supporting Information), respectively.

#### 2.2.1 o‐Ps Lifetime and Cage Filling

**Figure 1:**
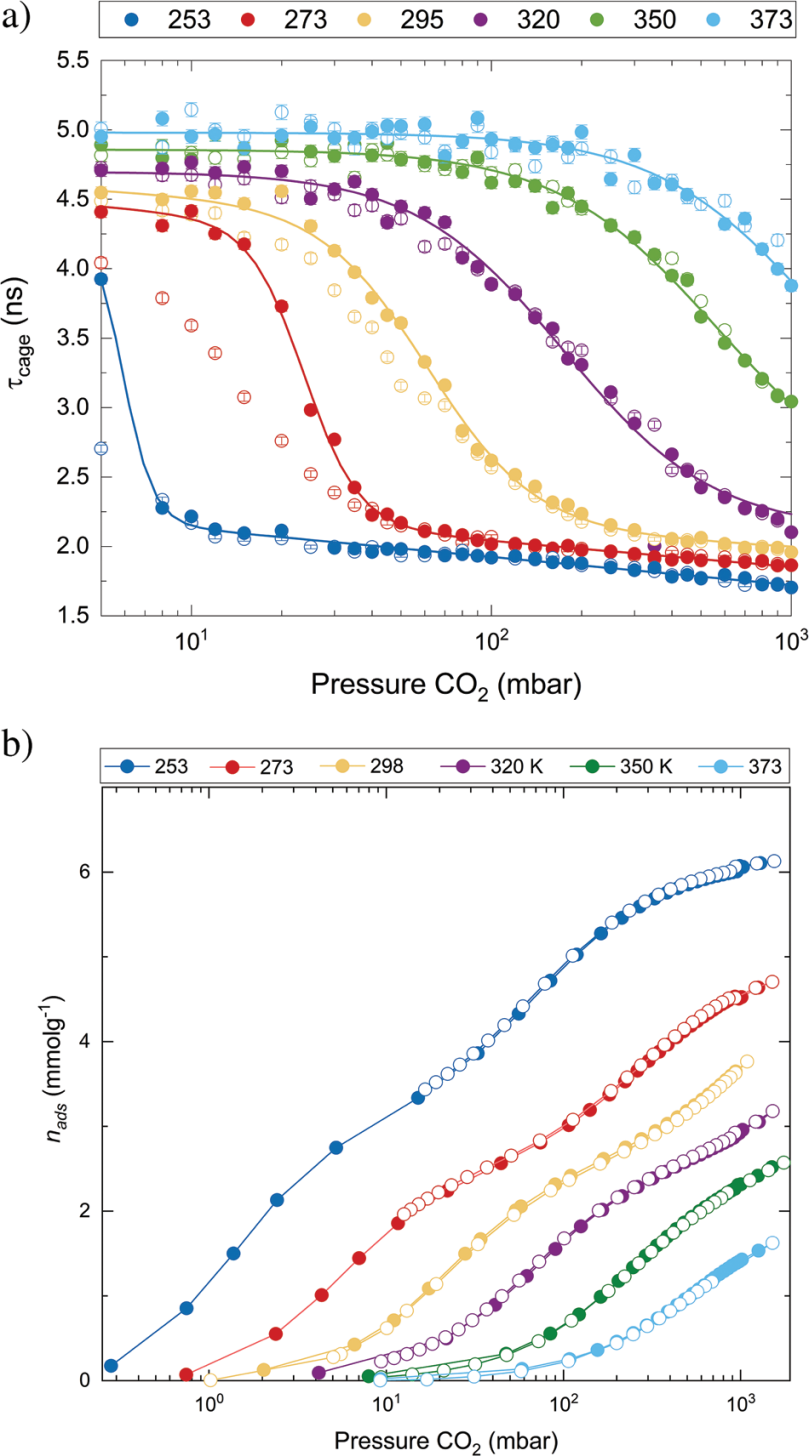
a) Pressure dependence of lifetimes of ortho‐positronium trapped in cages of CALF‐20 during CO_2_ sorption at different temperatures, where sigmoidal functions were fitted to adsorption data. b) Variable temperature CO_2_ physisorption isotherms in CALF‐20. Adsorption data are solid circles and desorption data are open circles.

As CO_2_ is introduced at different temperatures, significant changes occur in the o‐Ps lifetimes and intensities in the cages. For clarity, the entire course of changes can be best traced in the pressure range of 5–1000 mbar at room temperature (**Figure** **1**a and Appx. S3, Supporting Information). The change in *τ*_cage_ consists of three stages:

1. Lack of any change in lifetime below a certain pressure threshold value.
2. Sigmoidal decrease in lifetime that can be well described by equation
(1) $$\tau_{\text{cage}}=\frac{\tau_{\text{empty}}-\tau_{\text{filled}}}{1+e^{\frac{\ln\left(p/p_{1/2}\right)}{k}}}\;+\tau_{\text{filled}}$$

where *p* is CO_2_ pressure, *τ*_empty_, *τ*_filled_, *p_½_*, and *k* are fitting parameters meaning o‐Ps lifetimes before and after sigmoidal change, CO_2_ pressure at the middle of the sigmoidal change, and width of sigmoidal change, respectively.

1. Slow logarithmic decrease in *τ*_cage_ with *p*.

This pattern is observed at all studied temperatures; however, the sigmoidal change occurs at a very low pressure of several mbar at 253 K, while it spreads above the maximum CO_2_ pressure of 1000 mbar at 350 K and 373 K. Nevertheless, Equation 1 was fitted for all temperatures (Figure 1a and Table S1, Supporting Information). Given only the partial curves at *T* = 350 and 373 K, fitting was challenging. Therefore, we used fixed values from Figure S9 (Supporting Information) in all fits by extrapolating τ_filled_, assuming a linear change with temperature, and weighted the values with errors to ensure consistency.

**Figure 2:**
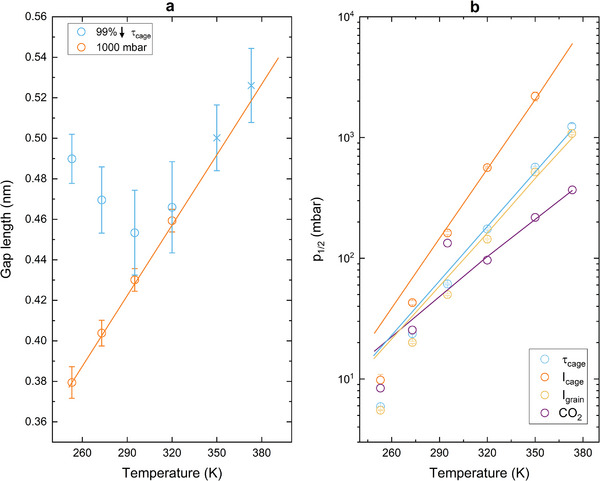
Temperature dependence of a) the length of the gap between CO_2_ molecules blocking the cages of CALF‐20 calculated from the o‐Ps lifetime assuming cage side obtained without gas when it is reduced by 99% of total change during the sigmoidal change and at 1000 mbar (circles), values for 350 and 373 K are extrapolated (crosses) and not applicable for 1000 mbar. b) CO_2_ pressure at which the fitted o‐Ps lifetime in cages (*τ*_cage_), fitted intensity of the cage (*I*_cage_) and intergranular (*I*_grain_) components, and CO_2_ uptake during the physisorption isotherm are halfway between the maximum values and the minimum values.

The decrease in o‐Ps lifetime in MOFs often results from complex interactions of o‐Ps with metal nodes.^[^ ^36^ ^]^ However, in CALF‐20 with Zn, where no unpaired electrons are present, there is no evidence of such effects—such as chemical quenching, spin conversion, Ps inhibition, or Ps complex formation. Instead, the predominating relevant Ps interaction is the pick‐off process, which is directly related to the size of the free volumes. Hence, the smaller lifetime implies varying degrees of cage filling with CO_2_ at different temperatures. Because the molecular diameter of CO_2_ (ca. 0.3 × 0.6 nm)^[^ ^43^ ^]^ is comparable with the size of the CALF‐20 cage, even a single CO_2_ molecule acts as a blockage of the pore and forms a potential barrier for o‐Ps. So, the o‐Ps probe detects an average distance between the blockages, which can consist of one or more molecules. In this context, the sigmoidal change observed in Figure 1a is the shortening of the distance between blockages from the infinity (open channel) to a certain minimum distance of Van der Waals radius due to filling cages with subsequent CO_2_ molecules. This distance between molecules can be calculated assuming a cuboid shape of free volume (**Figure** **2**a and Table S3, Supporting Information). At pressure just above the pore filling step in the *τ*_cage_ isotherm (when lifetime decreased by 99% of its total change) this gap is 0.45‐0.50 nm long, i.e., of a size that can accommodate one or two CO_2_ molecules. Below 298 K, where the step in *τ*_cage_ is observed at the pressure below 60 mbar further increase in pressure causes a reduction in the average gap length (*L*_gap_), which rate seems to be independent of temperature. Therefore, at 1000 mbar *L*_gap_ increases with temperature at a rate of 1.5 pm K^−1^ starting from 0.38 nm at 253 K (Figure 2a). These two effects, *L*_gap_ decreases with pressure and increases with temperature, reflect changes of the CO_2_ arrangement in the cages of CALF‐20. There is another possibility that o‐Ps exerts pressure on CO_2_ molecules, repels them, and thus *L*_gap_ increases. This effect, known as “bubble” formation, is observed in bulk liquids.^[^ ^44^ ^]^ However, as explained in the “*main cages and Ps bubble”* (Supporting Information), Ps bubble is not expected in the microporous CALF‐20 cages. Therefore, it is not justified to ascribe the changes in the gap size to the interaction of CO_2_ with o‐Ps.

The sigmoidal change related to cage filling with CO_2_ can be described by two parameters; i) *p_½_*, the CO_2_ pressure at which *τ*_cage_ is halfway from the maximum value (*τ*_empty_) to the minimum value (*τ*_filled_) when the *τ*_cage_ change is finished and ii) *k*, the dimensionless parameter describing the rate of the *τ*_cage_ change, where

- *p_½_* changes exponentially with temperature from tens of mbar at 253 K to over 1000 mbar at 373 K (Figure 2b).
- *k* increases linearly (i.e., width increases exponentially like *p_½_* because pressure in Equation 1 is introduced in the logarithmic scale, so for greater *p_½_* the same *k* value means greater width of the sigmoidal change) with temperature in the range of 253–320 K and then it seems to stabilize for higher temperatures (Figure S8, Supporting Information). It should be remembered that the sigmoidal change above 320 K is incomplete within the measured pressure range and these results can be inaccurate.

#### 2.2.2 Ps Intensities and Adsorption Mechanism

The intensities of both o‐Ps components undergo the sigmoidal change with increasing CO_2_ pressure. The intensity of the intergranular component (*I*_grain_) changes in almost the same way as the o‐Ps lifetime in cages (Figures S5 and S6, Supporting Information), following a function like that in Equation 1. The strong correlation between pressure dependences of fitted *τ*_cage_ and *I_grain_* (Figure 2b) confirms that of o‐Ps annihilating between grains originate from inside the cages. Filling the cages with CO_2_ results in blocking their outlets, preventing o‐Ps migration to the intergranular spaces. Possibly, at higher temperatures the mobility of CO_2_ increases and it can easily migrate to the center of the cage, leaving longer open ends that increase o‐Ps intensity outside grains when cages are filled (*I*_filled_ in Table S2, Supporting Information). This effect can be also related to the greater kinetic energy of o‐Ps at higher temperatures. Alternatively, *I*_grain_ in fact contains signals from o‐Ps in delocalized Bloch states, which spread over the periodic structure of CALF‐20 instead of localization in a single channel. In such a case, their formation and annihilation are affected by the presence of CO_2_ within the cages. This is thoroughly explained below.

**Figure 3:**
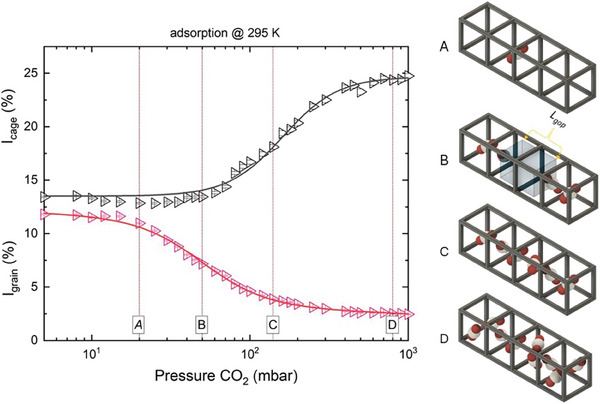
Left) changes of *I*_cage_ and *I*_grain_ as functions of adsorption pressure in CALF‐20 at 295 K. Right) Sketch illustrating the anticipated CO_2_ adsorption mechanism within CALF‐20 based on o‐Ps intensities.

More intriguing is the change in o‐Ps intensity in cages (*I*_cage_) with CO_2_ pressure (Figure S6, Supporting Information). One would expect it mirrors *I*_grain_, since o‐Ps, unable to escape the cages, will annihilate with the lifetime of *τ*_cage_ instead of *τ*_grain_. Although the expected increase in *I*_cage_ occurs and is approximately equal to the decrease in *I_grain_* (Figure S5, Supporting Information), it is clearly shifted towards higher pressures (Figure 2b). This shift ranges from almost two times greater pressure at 253 K to over three times at 320 K, and possibly more at higher temperatures, but this exceeds 1000 mbar and cannot be determined with sufficient accuracy. An additional channel of Ps migration outside grains is expected to justify high *I*_grain_ from a relatively large CALF‐20 crystals (Appx. S5 and Figure S12, Supporting Information). The origin of additional “intergranular” o‐Ps may be o‐Ps from delocalized (hot) Bloch states^[^ ^39^, ^45^ ^]^ spreading outside the grains, where disappearance of the periodic structure of CALF‐20 causes their localization. If this is the main source of *I*_grain_, its decrease is related to *τ*_cage_, reflecting a disruption of the periodic structure. In contrast, “intragranular” o‐Ps can hardly get outside grains and changes in *I*_cage_ are only slightly related to the migration of o‐Ps and reflect other processes in the cages that are hardly related to o‐Ps migration. The localized and delocalized o‐Ps states are discussed in the *“o‐Ps parameters during* in situ *CO_2_ adsorption”* (Supporting Information).

Notably, the changes in *I*_cage_ and *I*_grain_ over the pressure course aids in predicting the adsorption mechanism within CALF‐20. To explore the mechanism, we focus on their sigmoidal changes during CO_2_ adsorption. For clarity, we have selected the *I*_cage_ and *I*_grain_ curves at 295 K as a function of adsorption pressure and divided the sigmoidal part of *I*_grain_ (the fast‐changing parameter) into three sections; A, B, and C, while point D is assigned to represent the change beyond the sigmoidal transition (**Figure** **3**, left), to guide the discussion.

From vacuum until point A in Figure 3, left, both *I*_cage_ and *I*_grain_ are unchanged. Since o‐Ps intensity correlates to pore surface, hence the unchanged intensities suggest no pronounced change in inner cage surface. This indicates that few CO_2_ molecules are adsorbed at this pressure range and probably they locate in the center of the cage. As adsorption develops reaching point B (middle of the *I*_grain_ sigmoidal transition), *I*_grain_ declines but *I*_cage_ still unaffected, highlighting the shift mentioned above. Since the most probable binding sites of CO_2_ in CALF‐20 are in the middle of the cages, where the oxygen atoms of the oxalate groups in the framework serve as primary binding sites,^[^ ^19^ ^]^ the introduction of CO_2_ will disrupt the periodicity required to form the Bloch states and, therefore, reduces total Ps formation (Figure S7, Supporting Information), but only in the part that can localize outside grains (i.e., *I*_grain_). This initiate changes in *I*_grain_, while formation of Ps in the bulky part (*I*_cage_) is not affected and the shift is noticed. At this point, gaps between CO_2_ molecules are expected to get smaller inside the cage as *τ*_cage_ decreases (Figure 1a). When advancing to point C, additional CO_2_ molecules are introduced, which may form 1D chains along the channel. These chains further disrupt the periodicity of the cage. Consequently, the delocalized o‐Ps does not actually form. *I*_cage_ starts to increase because, in addition to electrons from CALF‐20, electrons from CO_2_ contribute more and more to the formation of o‐Ps. Finally, during stage D, *I*_cage_ reaches its maximum and *I*_grain_ approaches its minimum. In this region, *I*_cage_ increases by ca. 11.4%, while *I*_grain_ decreases by only 9.3% when compared to vacuum. The overcompensated increase in *I*_cage_ (only at 295 K and below) reveals an increase in the total o‐Ps formation (also seen in Figure S7, Supporting Information). This supports the argument that the changes in *I*_cage_ and *I*_grain_ originate from independent sources. The obvious cause of the increase in o‐Ps intensity is usually an increase in surface area. Hence, the increase of *I*_cage_ along stage D likely results from the filling of numerous free spaces with CO_2_, which not only occupies the central regions of the cages but also fits into the free spaces between the organic “pillars” connecting the metallic nodes in CALF‐20. Moreover, the increase in *I*_cage_ is not accompanied by an increase in *τ*_cage_, which can be explained if o‐Ps is trapped only in the central regions of the cages (between CO_2_ molecules) rather than in the smaller spaces between the “pillars.” This is justified because the smaller spaces between the pillars are connected to the larger cage centers, making it energetically favorable for o‐Ps to migrate from the smaller to the larger free spaces. Additionally, a shift of the CO_2_ molecules from the cage center to the cage sides at high pressures is also expected and it would lead to more empty spaces in the center that can host o‐Ps, which may also contribute to the increase in *I*_cage_ (stage D in Figure 3). The discussion about the adsorption mechanism based on the intensities is summarized in the sketch in Figure 3, right.

Detailed discussions about variations of the total o‐Ps intensity are provided in the “*o‐Ps intensity*” section in Appx. S3 (Supporting Information).

#### 2.2.3 Cage o‐Ps Intensity as a CO_2_ Uptake Indicator

As discussed above, Figure 1a illustrates the pore filling mechanism in CALF‐20, derived from in situ PALS data, and provides unique complementary information to the adsorption data (Figure 1b). In Figure 1b, the volumetric CO_2_ adsorption experiments at the same temperatures and pressure range reveal a Langmuir‐type isotherms with the uptake at 1000 mbar ranging from 1.4 mmol g^−1^ at 373 K to 6.0 mmol g^−1^ at 253 K. This shape of isotherm in Figure 1b and Figure S10 (Supporting Information), measured at the temperatures of PALS studies, shows an expected shape, which is in agreement with Shimizu and co‐authors.^[^ ^19^ ^]^ The isosteric heats of adsorption in Figure S11 (Supporting Information), calculated using Clausius‐Clapeyron equation^[^ ^46^ ^]^ overestimate the adsorption heat at low loadings (see Appx. S4, Supporting Information). At high loadings, the values are in range of 37–40 kJ mol^−1^, which is in line with earlier reported data.

**Figure 4:**
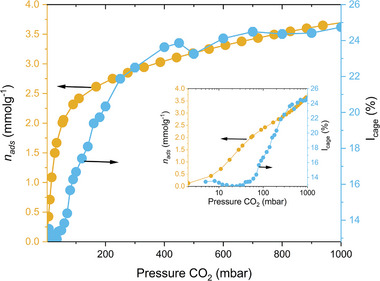
Experimental CO_2_ physisorption isotherm and variation of o‐Ps intensity in cages of CALF‐20 from in situ PALS during CO_2_ adsorption at 295 K. The same figure is plotted in the logarithmic scale in the inset.

Notably, the evolution of *τ*_cage_ and *I*_cage_ provide the complementary information on the pore filling mechanism indicating nearly complete pore filling before the saturation is reached. This is supported by the behavior of *p*_1/2_ in Figure 2b derived from fitting of *τ*_cage_ and *I*_cage_ and from CO_2_ physisorption, where it increases with temperature but with different rates. The *I*_cage_ (Figure S6, Supporting Information) directly correlates with the CO_2_ adsorption isotherm (Figure 1b) as shown in **Figure** **4** for isotherms at 295 K as an example. This encouraged us to explore the *I*_cage_ in more detail. The shape of *I*_cage_ resembles that of a type I isotherm,^[^ ^7^, ^19^ ^]^ indicating it reflects the amount of CO_2_ uptake. It would be beneficial to analyze if the change in *I*_cage_ can be traced by a known adsorption isotherm model. In Figure S6 (Supporting Information), *I*_cage_ exhibits a sigmoidal shape, which can be best described by the following equation:

(2) $$I_{\mathrm{cage}}=\frac{I_{\mathrm{empty}}-I_{\mathrm{filled}}}{1+{\left(\frac{p}{p_{1/2}}\right)}^{k}}+I_{\mathrm{filled}}$$

here, the parameters have the same meaning as in Equation 1. Equation 2 can be rearranged in the following form (derivation is presented in Equations S2–S5, Supporting Information):

(3) $$I_{\mathrm{cage}}=\frac{I_{\mathrm{filled}\;}\times \;R\;\times \;p^{k}}{1+R\;\times \;p^{k}}+\frac{I_{\mathrm{empty}}}{1+R\;\times \;p^{k}}$$

with *R* = ${\left(\frac{1}{p_{1/2}}\right)}^{k}$

Equation 3 can be regarded as a modified version of Langmuir–Freundlich (Sips) isotherm,^[^ ^47^ ^]^ which takes the form:

(4) $$q_{e}=\frac{q_{m}\times K_{s}\times C^{n}}{1+K_{s}\times C^{n}}$$

where *q*_e_ represents the amount of gas adsorbed at equilibrium, *q*_m_ is the maximum adsorption capacity, *K*_s_ is the proportional constant or Sips constant, *C* is the equilibrium gas concentration, and *n* is the dimensionless Sips exponent. By comparing Equations 3 and 4 we observe that *I*_cage_ follows the Langmuir–Freundlich isotherm with an additional term, $\frac{I_{\mathrm{empty}}}{1+R\times p^{k}}$. This term, involving *I*_empty_, likely arises from the formation of o‐Ps on the adsorption‐free sites on the walls of CALF‐20 at a given pressure. The terms in Equation 3 can be interpreted as representing CO₂ adsorption in the centers of the cages, along with the gradual filling of the open framework walls by CO₂ adsorbed. Additionally, the fitting exponent *k* in *I*_cage_ serves as a heterogeneity factor, similar to *n* in Sips isotherm. The reliability of the fitting parameters is shown in **Table** **2**. However, at 373 K, the lack of sigmoidal behavior in *I*_cage_ across all pressure values (Figure S6, Supporting Information) leads to unreliable fitting, and thus, the corresponding values have been omitted from Table 2 and other related figures.

**Table 2:** Fitting parameters obtained for the pressure dependence of *I*_cage_ at various temperatures *T* (Figure S6, Supporting Information): *p* is CO_2_ pressure, *p_½_* is the pressure at the middle of the sigmoidal change, *k* is the width of sigmoidal change, *I*_empty_ is intensity in cages without CO_2_, *I*_filled_ (*fixed on maximum value for all *T*) is intensity in cages with CO_2_.

T [K]	p_½_ [mbar]	Δp_½_ [mbar]	k	Δk	I_empty_ [%]	ΔI_empty_ [%]	I_filled_ [%]	ΔI_filled_ [%]
253	9.8	1.1	0.341	0.111	14.04	0.57	24.67*	0.14
273	42.9	1.7	0.246	0.036	13.79	0.49	24.67*	0.14
295	162.0	4.9	0.398	0.026	13.51	0.42	24.67*	0.14
320	563.1	8.5	0.496	0.015	13.19	0.37	24.67*	0.14
350	2198.7	187.3	0.628	0.045	12.81	0.37	24.67*	0.14

Although we illustrated that CO_2_ adsorption isotherm (as seen by *I*_cage_) adheres the Langmuir–Freundlich isotherm, Figure S11, supporting information and other studies have indicated that the CO_2_ adsorption is better described by a dual‐site Langmuir isotherm.^[^ ^7^ ^]^ Notably, the Langmuir–Freundlich isotherm accounts for heterogeneous adsorption sites, whereas the Langmuir isotherm assumes uniform adsorption sites. The fitted heterogeneity factor *k* for *I*_cage_ is depicted in Figure S8 (Supporting Information). It follows very closely the *k* values for *τ*_cage_ and *I*_grain_, which confirms that despite the pressure shift, change in these parameters result from the same effect. Additionally, it is important to highlight the difference between *I*_cage_ and *n*_ads_ in Figure 4 below 250 mbar (Figure 4, inset). In particular, below 50 mbar *I*_cage_ does not change with increasing *n*_ads_. This is possible if CO_2_ molecules are distant from the CALF‐20 skeleton, where thermalized positrons form o‐Ps at this stage, i.e., CO_2_ molecules are presumably located in the cage center. In the range between 50 and 250 mbar, this behavior gradually changes to the state where CO_2_ molecules start taking part in the o‐Ps formation, i.e., they locate in the neighborhood of the CALF‐20 framework more and more until both *I*_cage_ and *n*_ads_ follow the same dependence.

### 2.3 Humidity and Humid CO_2_ Adsorption

This section is focused on structural changes in CALF‐20 during CO_2_ adsorption in a humid environment, simulating CO_2_ capture under ambient conditions. The experiment was conducted at RT with 970 mbar of CO_2_ across relative humidity levels from 11% to 98%, and results were compared to CALF‐20 exposed to humidity alone. The 0% RH was realized by using molecular sieves to adsorb water from the sample chamber producing either dry air (reference for pure humidity experiment) or dry CO_2_ (reference for humid CO_2_ experiment). During the humid CO_2_ exposure, both humidity and CO_2_ were simultaneously introduced to the sample to mimic competition between H_2_O and CO_2_. The sample was reactivated in vacuum at 423 K for 4 h after each exposure, as detailed in the experimental section.

#### 2.3.1 Water Vapor Adsorption

*Adsorption*: Adsorption of water vapor on CALF‐20 sample at 298 K was conducted (Appx. S6 and Figure S13, Supporting Information (bottom)) in order to show the correlation between the PALS and adsorption data. The isotherm is similar to that reported by Shimizu and co‐workers.^[^ ^19^ ^]^ The adsorption branch indicates the steep water uptake in the pressure range *p*/*p*_0_ = 0–0.2. At higher pressure, less water is adsorbed in the pores. The desorption branch follows the adsorption showing a narrow hysteresis in the whole pressure range, originating from the slow adsorption kinetics of water vapor.

#### 2.3.2 Ps Components during Pure Humidity Exposure

**Figure 5:**
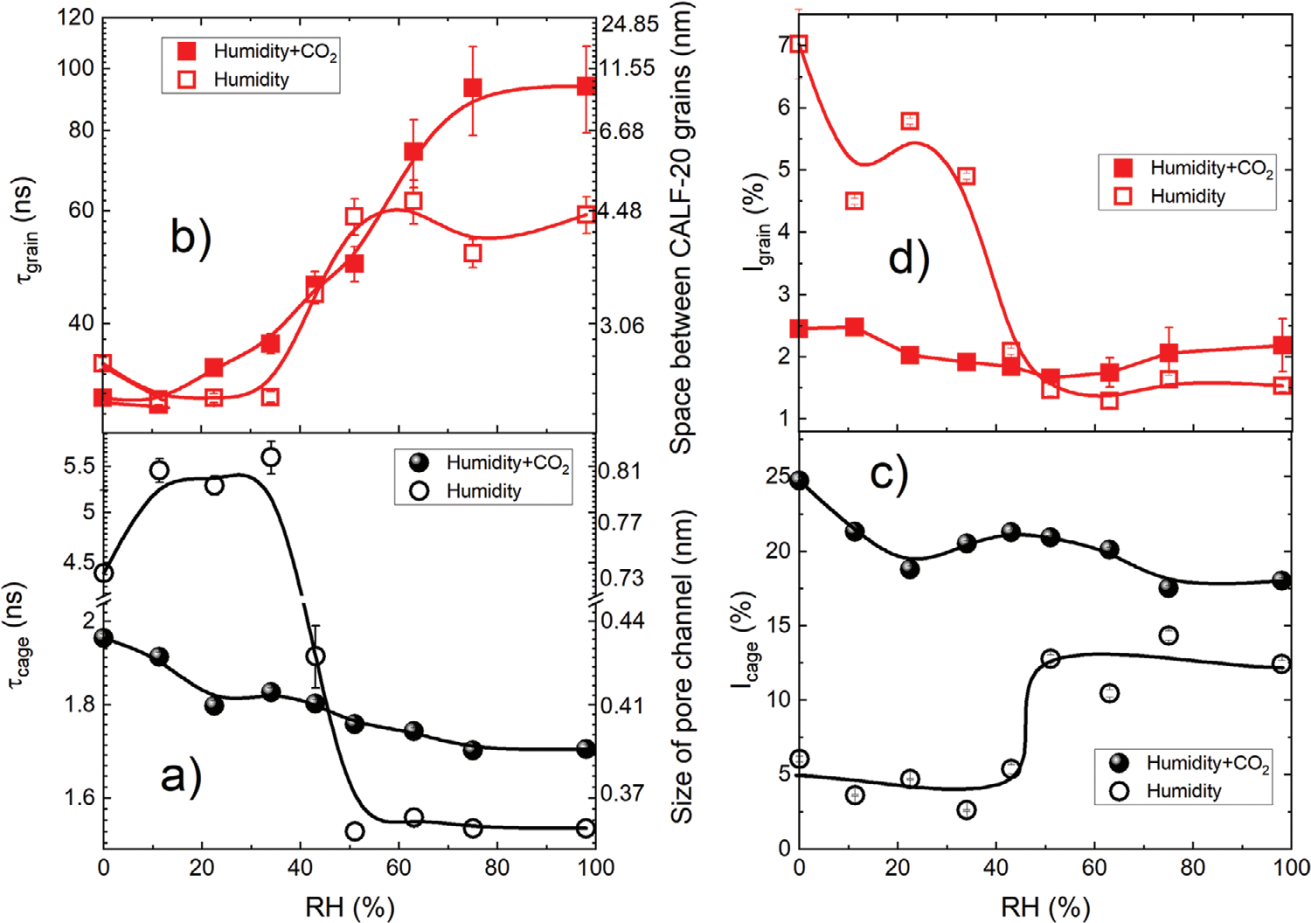
Impact of room temperature pure humidity exposure and 970 mbar CO_2_ adsorption in CALF‐20 as a function of relative humidity on: a) cage o‐Ps lifetime (*τ*_cage_)–right axis shows the derived size of the square cross‐section of cages, b) grain o‐Ps lifetime (*τ*_grain_), together with the corresponding size of gaps between calf‐20 grains (right axis), c) o‐Ps intensity in cages (*I*_cage_), and d) o‐Ps intensity between CALF‐20 grains (*I*_grain_ *)*. Lines are shown to guide the eye.

*Cages*: In the pure humidity run at RT, a step increase in *τ*_cage_ of 1 ns between 0% and 11% RH is observed (**Figure** **5**a). The decrease in o‐Ps pick‐off annihilation rate (increase in lifetime) shows that water somehow screens the interaction between o‐Ps (already formed in the bulky parts) and the electrons on the pore walls. This suggests that, unlike CO_2_, adsorbed water is initially located not in the center but closer to the cage's skeleton. This observation is supported by the snapshots in ref.[48] and the shortest distances between the guest molecules (CO_2_ or H_2_O) inside the cage and the CALF‐20′s skeleton in refs.[19, 49] For H_2_O, the shortest distance was estimated to be 2.8 Å using radial distribution function (RDF),^[^ ^49^ ^]^ whereas for CO_2_, it was calculated to be 3.03 Å through atomistic grand canonical Monte Carlo simulations.^[^ ^19^ ^]^ After that, CALF‐20 exhibits two distinct regions of τ_cage_ with 35–50% RH as a transition region (Figure 5a). Below 35% RH, τ_cage_ fluctuates around ≈5.5 ns. As noted by Oktavian et al.,^[^ ^49^ ^]^ the RDF analysis suggests that at low RH, water molecules position themselves near the oxygen atoms of the oxalate linkers and further away the Zn atoms, resembling a hydrophobicity. Water molecules thus prefer direct cooperative contact, forming zero or at most one hydrogen bond, resulting in isolated water molecules or small oligomers being adsorbed.^[^ ^22^ ^]^ This minimal hydrogen bond concentration at low RH slightly affects *τ*_cage_, with its variability screening o‐Ps interactions with the few water molecules present in the cages. Above 35% RH, τ_cage_ shows an abrupt reduction to ≈1.5 ns between 35% and 50% RH and stabilizes afterwards at this value regardless of the RH level. The steep transition in τ_cage_ between 35 and 50% RH may indicate the onset of cage filling, aligning with water isotherm in Figure S13 (Supporting Information) (bottom), which starts to saturate at almost the same values of relative pressure. However, the τ_cage_ value of 1.5 ns at RH > 50% is shorter than the o‐Ps lifetime of 1.87 ns in bulk water (Ps bubble) or 2.15 ns in water confined in SBA‐3 with 2 nm pores.^[^ ^30^ ^]^ The lifetime of 1.5 ns corresponds to the residual water‐free spaces with size of ca. 0.3 nm that are present in the cages.^[^ ^27^ ^]^ These water‐free spaces are expected because studies have demonstrated that at higher RH levels, water form complex hydrogen‐bonding networks and quasi‐1D molecular chaines within the MOF cage,^[^ ^26^, ^50^ ^]^ and spaces may exist between water chains.

Between 0% and 35% RH, *I*_cage_ (Figure 5c) gently decreases reaching 3% at 35% RH. Like the discussion of *τ*_cage_, this reduction in *I*_cage_ may originate from the low amount of adsorbed water. Above 35% RH, *I*_cage_ starts to rise, reaching 12–15% at RH ≥ 50%. According to Llewellyn et al.,^[^ ^22^ ^]^ at high values of partial pressure *p*/*p*_0_ (∝ RH), percolation occurs, causing neighboring water chains to interconnect. These interconnected water chains begin to form clusters around the inner pore surface,^[^ ^22^ ^]^ potentially creating spaces in between. This increase in the surface area along with detachment of water molecules from walls, which gives o‐Ps an opportunity to repel them, could explain the observed growth of *I*_cage_.

*Inter‐Grain Spaces*: τ_grain_ in Figure 5b in the absence of CO_2_ also exhibits a three‐region dependence on RH, i.e., 0–35%, 35–50%, and 50–98%. At RH below 35%, τ_grain_ slightly decreases, likely due to the presence of some water molecules in the intergranular spaces, but mostly in the cages, where they affect the delocalized o‐Ps reducing its intensity and, in consequence, the average lifetime of migrated and delocalized o‐Ps. Next, it increases linearly up in the RH range between 35% and 50%, and then stabilizes around 60 ns. This increase in τ_grain_ indicates an enlargement of the spacing between CALF‐20 layers or grains. This might be a result of enhanced structural flexibility, leaving larger spaces between grains, but more likely water condenses (supported by the increasing branch of water adsorption isotherm in Figure S13, Supporting Information) at high humidity level and fills the smaller spaces in the grain spaces leaving the larger spaces unfilled. This can lead to a longer lifetime for o‐Ps annihilating in the residual larger spaces. Alternatively, the suppression of re‐migration of o‐Ps to the cages is responsible for the increase in τ_grain_, which is described in detail in the “*Humidity and Humid CO_2_ in CALF‐20”* (Supporting Information).

*I*_grain_ drops between the dry state (0% RH) and the first humidity point (11%). This drop is not compensated in *I*_cage,_ and it indicates lower o‐Ps formation as previously discussed. Again, this would mean that the inner pore surface is altered and the number of available sites to form the delocalized o‐Ps becomes less. Subsequently, *I*_grain_ varies between 4% and 6% at RH ranging from 11% to 35% (Figure 5d), likely due to some adsorbed water molecules between grains. At RH = 43%, *I*_grain_ falls sharply to about 2%, and continues to decrease to 1.2–1.5% at higher RH levels. Aligning with the discussion of τ_grain_, this abrupt reduction in *I*_grain_ can be attributed to the presence of residual larger intergranular spaces, while the smaller intergranular spaces, which constitute the majority, have already been filled.

#### 2.3.3 Ps Components during Humid CO_2_

*Cages*: In this part, CO₂ at 970 mbar is introduced together with humidity at RT. The 970 mbar pressure was specifically chosen to observe the most pronounced effects on the material's porosity and adsorption dynamics by PALS. While this pressure exceeds typical environmental conditions, it enhances the differentiation of changes in o‐Ps lifetimes and intensities, thereby facilitating a clearer interpretation of the underlying adsorption mechanisms. This approach provides insights that can later be extrapolated to more realistic conditions at lower CO₂ pressures. Once the RT humid CO_2_ is applied, *τ*_cage_ decreases, but with distinct characteristics. Initially, it drops from 1.95 to 1.8 ns between 0 and 40% RH (Figure 5a). The corresponding cage size changes from 0.43 to 0.41 nm. Although it has been noted that humid CO_2_ induces a phase change from α‐CALF‐20 to β‐CALF‐20 above 23% RH,^[^ ^20^ ^]^ from Ps lifetime we cannot unambiguously attribute the change in cage sizes solely to this phase change due to the progressive cage filling. In humid CO_2_ at low RH, the uptake of CO_2_ is favored,^[^ ^19^ ^]^ but water molecules coexist. The oxalate groups in the framework serve as primary binding sites for both H_2_O and CO_2_;^[^ ^26^ ^]^ however, their spatial distribution within the pores differs^[^ ^19^, ^49^ ^]^ as stated earlier, i.e., H_2_O molecules are located near the framework's skeleton, which possibly due to variations in molecular interaction of CO_2_ and H_2_O with the framework, interaction strengths, etc. Consequently, there will be a competitive interaction between H_2_O and CO_2_ in CALF‐20, due to their shared preference for the same void regions and similar interaction energies with the framework. This competitive adsorption phenomenon at low RH is consistent with the recent findings of Krishna and van Baten,^[^ ^51^, ^52^ ^]^ who demonstrated through molecular simulations that CO_2_ molecules face reduced competition than expected from uniform adsorption models, such as the ideal adsorbed solution theory (IAST) due to the segregated adsorption of H_2_O, highlighting the preference of CO_2_ uptake at low RH. Worth noting, recent work by Moreton et al.^[^ ^53^ ^]^ of varied temperature (298 K, 308, and 318 K) CO_2_–H_2_O mixture adsorption on CALF‐20 thin films have illustrated that the deviation from IAST theory is only pronounced at RT, while it is of less importance at higher temperatures. Aligning with the work of Krishna and van Baten,^[^ ^51^, ^52^ ^]^ the *τ*_cage_ in Figure 5a at low water content (up to 40% RH) suggests that the interaction between water and CO_2_ (or oppositely—the interaction screening) may promote partial pore filling in addition to a possible phase change. This implies that the blockages created by CO_2_, as described previously, are not entirely closed, allowing water molecules to intrude between them. Indeed, RDFs and computational snapshots in refs. [51, 52] confirm that H_2_O–H_2_O pairs are typically located between CO_2_ molecules at distances of approximately 3 Å, while CO_2_–H_2_O pairs are spaced farther apart at around 8 Å, and CO_2_–CO_2_ separations are approximately 6.5 Å. In this context, *τ*_cage_ serves as a probe for the average empty spaces within the pores. This suggests that within the pores, water molecules can rapidly exchange between the cages without adhering to each other or to the pore walls because the pores are very small. Due to the co‐adsorption, H_2_O occupies energetically favorable positions, which appear to be situated between the CO_2_ molecules. Therefore, the distinguishability between the phase transition and pore filling cannot be made with a reasonable precision. An increased tendency for water molecules to form hydrogen bonds among themselves was observed at RH > 40% in CALF‐20.^[^ ^19^, ^20^, ^22^, ^26^ ^]^ Consequently, more interconnected hydrogen‐bond networks are formed involving a greater number of H_2_O molecules. The strong hydrogen bonding for H_2_O–H_2_O pairs at high RH may lead to a substantial increase in the competition between CO_2_ and H_2_O, as also corroborated by Krishna and van Baten.^[^ ^51^, ^52^ ^]^ They reported that under these conditions, CO_2_ molecules are forced to share adsorption sites with chains of hydrogen‐bonded H_2_O molecules appearing at distances of about 3 Å, leading to a significant reduction in CO_2_ uptake and further deviation from IAST predictions. Hence, we hypothesize that the formation of water chains, along with the increased preference for water uptake over CO_2_ at high RH levels, is manifested in the reduction of τ_cage_ to approximately 1.8 ns.

The presence of both CO_2_ and humidity leads to smaller, but distinct, changes in *I*_cage_ than in pure water vapor indicating that the micropores are permanently filled with the guests. *I*_cage_ is lower than in the dry CO_2_ at 970 mbar (0% RH)—due to less Ps formation because of changes in the chemistry of inner cage surface when water exist, but it hardly changes with humidity up to ca. 70%. Above this pressure (at 10^3^ mbar at 295 K in Figure S6, Supporting Information) it still remains clearly above the humidified sample. This confirms our previous interpretation that water molecules are initially well dispersed in the cages filled with CO_2_ and only start to cluster at high humidity.

*Inter‐Grain Spaces*: In the presence of humidity together with CO_2_, τ_grain_ behaves slightly different than in pure water adsorption experiment. When humidity and CO_2_ coexist, *τ*_grain_ starts changing already at 23% RH and increases linearly with RH until stabilizing at ca. 90 ns between 75% and 98% RH (Figure 5b). The transition differs from pure humidity not only due to the wider RH range, but also the different slope in *τ*_grain_ changes and much greater value at high RH. It seems that the intergranular spaces are much larger at RH > 75%. The increased spaces may imply structural flexibility; however, the current results alone do not provide strong evidence to support this. Alternatively, different interactions between the humid gas and the delocalized o‐Ps migrating to intergranular spaces may alter the ratio of o‐Ps re‐entering the cages. This results in fewer o‐Ps annihilating with the cage lifetime, while more o‐Ps remain confined in the grain spaces, where they annihilate with longer lifetimes due to the larger size of these spaces.

In humid CO_2_ adsorption, *I*_grain_ in Figure 5d starts at a low level to decrease with increasing RH until 50%. This decline may result from both hindered migration of o‐Ps out of the cages and alterations in the surface chemistry of the outer particle surfaces facing the grains. It remains greater than in the humid sample without CO_2_ at higher RH. This again could mean that more o‐Ps are being confined in intergranular spaces due to the humid gas, where CO_2_ accumulating between the grains, as water preferentially fills the cages at high RH. This is supported by *I*_grain_ at RH > 70% approaching its values observed in dry CO_2_ (at 0% RH). However, positronium formation may be affected by other than geometrical factors, e.g., a high probability of Ps formation on the surface of water clusters or even near to their surface (if Ps can migrate outside to the inter‐grain spaces).

### 2.4 Variable Temperature PXRD in the Controlled Atmospheres

**Figure 6:**
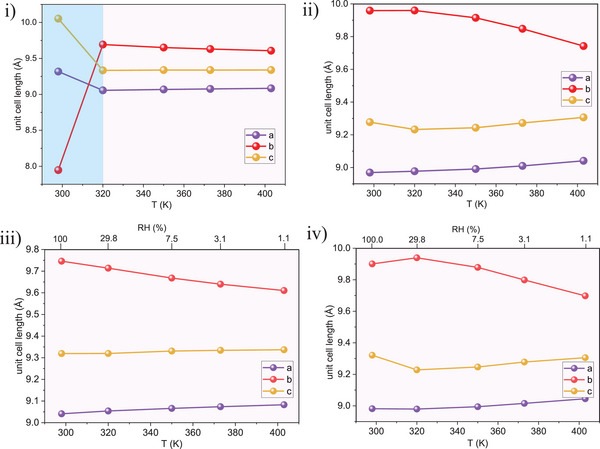
Unit cell axes evolution in CALF‐20, measured at different temperatures in i) dry nitrogen; ii) dry carbon dioxide; iii) wet nitrogen; iv) wet carbon dioxide (conditions for α‐CALF‐20 are labelled yellow, β‐phase light blue).

To confirm the phase composition of the pristine sample at room temperature and under various temperatures and gas atmospheres explored by PALS and CO₂ physisorption, PXRD patterns were collected for the CALF‐20 sample (Appx. S7 and Figure S14, Supporting Information), which was exposed to dry and humid nitrogen and carbon dioxide gases. All PXRD data were analyzed using Pawley method, and the results are shown in **Figure** **6**. The first measurement, conducted on the CALF‐20 sample at 298 K in a dry nitrogen flow and prepared under ambient conditions, indicates the presence of the pure CALF‐20‐β phase (Figure 6i). Increasing the temperature to 320 K induces a phase transition from CALF‐20‐β to CALF‐20‐α, primarily reflected by an expansion along the b‐axis and simultaneous contractions along the a‐ and c‐axes. This finding is significant as it suggests that desorbing water and purging the CALF‐20 cages require minimal energy, in accordance to observations of Chapman et al.^[^ ^20^ ^]^ in their helium flow experiments at room temperature. At higher temperatures, only the CALF‐20‐α phase was maintained. These results also support the PALS findings in Table 1, where the CALF‐20‐α phase was observed at T > RT. In the case of wet nitrogen flow, only the CALF‐20‐α phase was observed across the entire temperature range (Figure 6iii). Notably, the observed variations in unit cell parameters are consistent with in silico data previously reported by Maurin et al.^[^ ^54^ ^]^ When exposed to dry CO₂, no phase transition was initiated, and the CALF‐20‐α phase was observed across the entire temperature range (Figure 6ii). Interestingly, wet CO₂ also did not induce a phase transition, even at 298 K,^[^ ^54^ ^]^ indicating strong host‐guest interactions between CO₂ and the CALF‐20‐α phase (Figure 6iv).

The data obtained provide essential insights into the behavior of CALF‐20 under various conditions, specifically:

- Under ambient conditions (298 K and 40–60% RH), CALF‐20 exists in its β‐phase (smaller pores, partially filled with water);
- At elevated temperatures (320 K and above), CALF‐20‐β transitions to CALF‐20‐α, regardless of the gas type or humidity;
- At ambient temperature in the presence of pure CO₂, interactions between CO₂ and CALF‐20‐α are stronger compared to water, resulting in no observed phase transition.

## 3 Conclusions

This study provides comprehensive insights into the thermal effects on the porosity of CALF‐20(Zn) under dry and humid CO_2_ adsorption conditions, emphasizing the complex interplay between temperature, humidity, and guest interactions. In situ‐PALS data reveal that o‐Ps lifetimes in CALF‐20 vary from 253 K to 373 K, with only a modest 4% increase in cage size under vacuum, indicating that structural integrity is preserved for effective CO_2_ adsorption.

The analysis shows a sigmoidal change in o‐Ps lifetimes and intensities during CO_2_ adsorption, highlighting the material's responsiveness to temperature and pressure variations. Notably, PALS results indicate the CO_2_ adsorption results in some empty spaces or gaps inside the CALF‐20 cages, that are temperature‐ and pressure‐dependent. The correlation between PALS measurements and volumetric CO_2_ adsorption confirms that CALF‐20 exhibits Langmuir‐type isotherms in the studied temperature range.

In humid environments, the competitive interactions between water and CO_2_ significantly affect the course of adsorption on CALF‐20. Depending on the humidity, three clearly distinct states of the system can be observed: dispersed water molecules, transition state, and water clustering. Above the transition, the formation of interconnected water chains around the inner pore surfaces is observed, along with possibly enhanced structural flexibility and larger inter‐grain spaces filled with condensed water. When CO_2_ is introduced alongside humidity, the size of adsorbate‐free volumes in the cages initially decreases but stabilizes as hydrogen‐bond networks form among water molecules at high RH. This indicates competitive interactions for binding sites within CALF‐20′s framework. Variable temperature PXRD confirms that CALF‐20 transitions from β‐phase to α‐phase at elevated temperatures (320 K and above), independent of gas or humidity conditions.

Overall, the integration of PALS, gas and water physisorption, and in situ PXRD analyses in this work elucidates the fundamental mechanisms governing CO_2_ adsorption in CALF‐20 while underscoring the importance of environmental factors in optimizing its application for carbon capture technologies. Future research should focus on long‐term stability and performance under varied operational conditions to fully reveal CALF‐20′s potential.

## 4 Experimental Section

### Materials

CALF‐20 powder was purchased from Sigma Aldrich and used in adsorption experiments, in situ‐PXRD and in situ PALS measurements after desolvation in dynamic vacuum for 10 h at 430 K.

### Methods—PALS: Data Collection and Treatment

PALS measurements were conducted using a digital positron lifetime spectrometer equipped with an Acqiris DC 282 digitizer (10‐bit vertical resolution) and three photomultiplier tubes (PMTs) from SCIONIX HOLLAND coupled to CeBr_3_ scintillators. Two scintillators had dimensions of Ø = 51 mm and *h* = 10 mm, while the third scintillator had *Ø* = 51 mm and *h* = 25.4 mm. The scintillators were deliberately positioned to ensure comprehensive coverage around the sample holder and minimize the detection of backscattered photons, which can distort PALS spectra due to high efficiency of scintillators. The PMTs were arranged with precision: two were oriented horizontally at a 90° angle to each other, with their front faces separated by approximately 20 mm. The third PMT was vertically aligned, positioned above the sample holder. Customized data acquisition software facilitated simultaneous detection of start and stop signals from each of the three PMTs. This capability allowed for the collection of six spectra in a single measurement session, significantly reducing measurement time compared to traditional two‐tube spectrometers. The sample holder was designed specifically to accommodate around 0.4 mL of powdered sample, surrounding a 20 µCi ^22^Na positron source sealed within a 5 µm Kapton foil (DuPont). The coincidence box was set to a time range of 500 ns, while the PALS data acquisition software operated with a 600 ns time window and a channel width of 5 ps. The PALS spectra were resolved by fitting exponential decay curves to the histograms of recorded time differences, revealing lifetime components (*τ*_n_) with their respective intensities (*I*_n_). Analysis showed that a reliable fit, characterized by minimal residual and a *χ* ^2^ value of 1.03–1.08, was achieved only when considering four lifetime components. The resolved lifetime components were categorized based on their lifetimes: τ_1_, corresponding to p‐Ps annihilation (τ_1_ ≈ 0.12–0.18 ns); τ_2_, originating from unbound e^+^ annihilation (τ_2_ ≈ 0.35–0.50 ns); and the longest‐lived components representing o‐Ps annihilation within the pores (τ_3_ > 1 ns). It is believed that τ_4_ reflects o‐Ps annihilation between crystals in the intergranular spaces. These components are visualized in Figure S2 (Supporting Information).

The numerical spectra analysis was conducted using the PALSFit routine,^[^ ^42^ ^]^ which separates the spectra from resolution functions, background, and source contribution. The conversion of the measured o‐Ps lifetime to pore sizes is done by the EELViS code,^[^ ^55^ ^]^ which is based on the extended Tao‐Eldrup model.^[^ ^40^ ^]^ Al and Sn reference samples with known lifetimes were employed to estimate the resolution functions and source contribution. Approximately 10.6% source contribution (distributed between Kapton foil (0.382 ns /10.4%) and glue (2.6 ns/0.2%)) and an average time resolution of 0.28 ns (full width at half maximum) were determined during the fitting process.

### In Situ PALS Measurement Methodologies

The utilized PALS setup coupled to a gas dosing system is detailed elsewhere.^[^ ^41^ ^]^ Whenever no pressure or temperature scanning is performed, the PALS measurements were conducted in slices with a duration of 2 h per slice (required to accumulate ≈3 × 10^6^ annihilation events) to monitor the attainment of equilibrium conditions over the sample, such as humidity saturation, and to ensure system stability over time. The results presented in this study represent the average of the slices, as indicated below. The measurement sequence included:

- Activation: The as‐received sample underwent activation at 423 K in five slices (totaling 10 h, with 2 h per slice) under dynamic vacuum of 1.5 × 10^−6^ mbar to remove any impurities absorbed by the CALF‐20 during sample transfer for PALS measurements. Subsequently, the evacuated sample was cooled to room temperature (RT) and PALS spectrum was measured again over 2 h. This RT measurement serves as a reference for the initial pore state.
- Temperature‐dependent measurements of empty pores: As mentioned in Section 2.1, PALS measurements were performed under vacuum conditions (1.5 × 10^−6^ mbar) over a temperature range of 253 to 373 K. The experimental procedure involved first activating the sample at 423 K for 10 h, followed by a temperature program starting at 373 K and cooling to 253 K, with heating and cooling rates of 2.5 K min^−1^. Measurements were conducted at 2 h intervals at each temperature step during the temperature program.
- Dry CO_2_ adsorption: The dry CO_2_ adsorption experiments have been carried out at different temperatures: 353 K, 323 K, 295 K, 273 K, and 253 K. At each T, the gas pressure was regulated by the software and varied from 5 mbar to 1000 mbar during the adsorption cycle and then reversed during the desorption cycle. The PALS measurements for each adsorption/desorption step were conducted continuously for 2 h at specific temperature and gas pressure conditions. After each adsorption scan at a specific temperature, the sample was annealed at 473 K for 4 h to purge the pore for the subsequent step.
- Humidity and humid CO_2_: Again, to thoroughly remove any residual CO_2_ in the pores from the previous experiment, the sample underwent reactivation at 423 K for 4 h. Following this, the humidity experiments commenced at RT, consisting of two steps: first, exposing the sample to pure humidity, and then subjecting it to humid CO_2_ conditions at 970 mbar CO_2_. Humidity levels were adjusted using saturated salt solutions to achieve specific relative humidity (RH) levels of 11.3%, 22.5%, 34%, 43%, 51%, 63.5%, 75%, and 98% (water). At each RH value, the procedure involved closing the valve to CO_2_ to establish pure humidity, removing the salt solution, heating the sample under vacuum at 423 K for 4 h to restore clean pores, reintroducing the salt solution, and simultaneously opening the CO_2_ valve to initiate the humid CO_2_ experiment. Subsequently, the sample was left exposed overnight to ensure saturated humidity over the sample. The measurement then carried out by recording at least five slices, each lasting 2 h, and the average of these slices is presented as the result. Then, the activation procedure was repeated before the next step.

### Methods—Volumetric Adsorption Measurements

Water vapor physisorption isotherms were measured using BELSORP‐max apparatus (MICROTRAC MRB). A water‐filled thermostat combined with water bath (*T* = 298 K) was used to control the temperature. Helium gas (purity 99.999%) was used for dead volume measurement. The conditions of 0.5% of pressure change within 450 s was assumed as adsorption equilibrium. Carbon dioxide physisorption isotherms were measured using BELSORP‐HP (MICROTRAC MRB), equipped with closed cycle helium cryostat and customized adsorption cell. The dead volume was measured using helium gas (purity 99.999%) and carbon dioxide (purity 99.999%) were used in adsorption experiments. The adsorption equilibration was defined as 1% of the pressure change within 450 s.

### Methods—Variable Temperature PXRD in Controlled Gas Atmospheres

Powder diffraction patterns at variable temperatures were measured on PANALYTICAL Empyrean Powder X‐ray diffractometer (α‐1 system, ω‐2θ goniometer), equipped with Cu‐Kα1 radiation (primary monochromator *λ* = 1.54059 Å), motorized antiscatter and receiving slits. A Pixcel 1D detector was used for the measurements of reflection intensities. HTK‐1200N high temperature chamber (ANTON PAAR) was used to control the sample temperature. All measurements were conducted in a gas flow of 5 mL min^−1^. PXRD patterns were collected in reflection geometry using ω‐2θ scans in the 2*θ* range from 3 to 70° with 0.013° steps and 120 s per step. The sample was conditioned for 30 min after reaching the target temperature. The sample height correction program for the measurements in the inert atmosphere was applied to correct the thermal expansion of the sample holder. Nitrogen (purity 99.999%) and carbon dioxide (purity 99.999%) were used in experiments. The gases were humidified in a customized water‐filled bubbler in order to achieve a saturated humidity level at 298 K.

## Conflict of Interest

The authors declare no conflict of interest.

## Supporting information



Supporting Information

## Data Availability

The data that support the findings of this study are available from the corresponding author upon reasonable request.
