# Supplementary material for: Uncovering the Dynamic CO2 Gas Uptake Behavior of CALF‐20 (Zn) under Varying Conditions via Positronium Lifetime Analysis
Source: Small. 2025 Feb 25;21(14):2500544. doi: 10.1002/smll.202500544 (PMC11983255; doi:10.1002/smll.202500544)
Supplement: Supplementary file 1 — Supporting Information [file SMLL-21-2500544-s001.docx]

Supporting Information

**Uncovering the Dynamic CO_2_ Gas Uptake Behaviour of CALF-20 (Zn) under Varying Conditions via Positronium Lifetime Analysis**

*Ahmed G. Attallah*, Volodymyr Bon, Eric Hirschmann, Maik Butterling, Andreas Wagner, Radosław Zaleski*, Stefan Kaskel**

**Appendix S 1. Positronium: a probe of nanoscopic free volumes**

Positions (e^+^), emitted from ^22^Na source with energy endpoint of 540 keV, interact with matter, ionizing and exciting atoms until they reach thermal energy, at which point e^+^ diffuse throughout a material. In materials with free volumes, such as MOFs, thermalized e^+^ can bond to one of the ionized electrons (e^-^) to create the positronium (Ps) atom, according to the spur model.^[1]^ The relative spin alignment of e^-^ and e^+^ determines which of the two states the Ps atom exists: a spin antiparallel singlet state, known as para-Ps (p-Ps), or a spin parallel triplet state, known as ortho-Ps (o-Ps). Because p-Ps self-annihilates with a typical lifetime of 0.125 ns and is hardly affected by pore size or medium, it cannot be utilized to assess porosity. However, PALS porosimetry focuses on the lifetime of o-Ps.^[2]^ In vacuum, o-Ps would annihilate with its paired electron, but in material environments, it’s susceptible to interactions with electrons from the atoms on the walls of the pores, particularly those with spins opposite to the positron in the o-Ps. This interaction, known as *pick-off* annihilation,^[3]^ is crucial in determining the lifetime of o-Ps within PALS porosimetry. The probability of pick-off annihilation is related to pore size; in smaller pores, the annihilation rate is higher due to increased overlapping of o-Ps wavefunction with the walls, leading to a pore size-correlated o-Ps lifetime. The Tao-Eldrup (TE) model is employed to convert the fitted o-Ps pick-off lifetimes into micropore radii (R).^[4,5]^ The Tao-Eldrup model modifies a spherical pore, typically described by a rectangular potential well with a radius R and a finite potential barrier, by replacing it with a well that has an infinite potential barrier and a dimension of R + Δ. This adjustment allows to reproduce overlapping of the wavefunction Ψ(r) of the o-Ps in the ground state of the finite potential well with the bulk material by the wavefunction in the infinite potential well, assuming the electron layer within the pore wall from R to R + Δ. In this model, Δ serves as an empirical parameter. The o-Ps pick-off annihilation rate is then equal to the annihilation rate in the bulk multiplied by the probability of finding the Ps outside the potential well. This probability is calculated by integrating |Ψ(r)|² * r² from R (the boundary where the electron density begins to be non-zero) to R + Δ. It is important to note that the TE model is only applicable to micropores. In these small pores (narrow potential wells), the energy level spacing is large enough to prevent o-Ps from occupying higher energy levels. However, in larger pores, excited states must be considered. As a result, the TE model is adapted for larger pore sizes and different pore geometries through extended TE models (ETE).^[3,6]^

**Appendix S 2. Pore size distribution of CALF-20 pores from crystallography**

Comparing the derived d_cage_ (Table **1**) with the theoretical pore size distribution calculated from PXRD by Zeo++ (Fig. **S1**) reveals that the CALF-20 cages exist in the α phase.


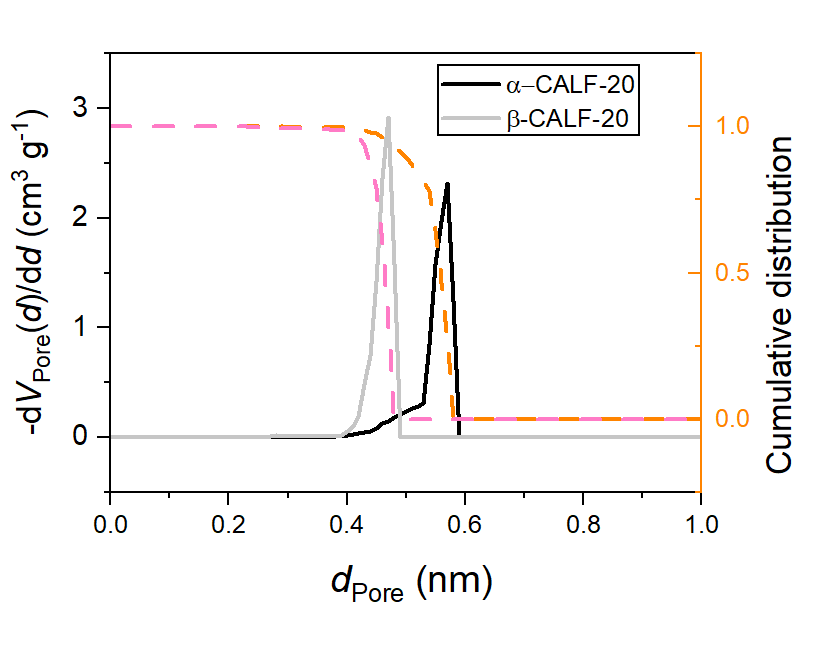


**Figure S 1**. Theoretically calculated pore size distribution for α- and β-CALF-20.

**Appendix S 3.** **PALS results**

***Ps components.***

PALS spectra, which follow the principles of radioactive decay, are composed of multiple exponential decay components. These components are related to different positron fractions: free positrons, p-Ps, and o-Ps, each with its own characteristic lifetime (the inverse of the decay rate that is proportional to the curve slope) and intensity (the area under the curve). The distinct lifetimes and intensities of these components provide valuable information about the material's microstructure and porosity. In CALF-20, the analysis of positron annihilation histograms indicated the presence of four distinct lifetime components (Fig. **S2**). The origin of these components is discussed in the method section and in Fig. **S2**. Worth noting, that in the absence of chemical reactions, o-Ps intensity can serve as a qualitative indicator of pore concentration. This means it can be used to monitor changes in pore concentration in response to external parameters, such as varying gas pressure.

***o-Ps parameters during in situ CO_2_ adsorption***

***Intergranular spaces***

In positron porosimetry, the component with a lifetime of tens of nanoseconds (Fig. **S3**) is usually ascribed to


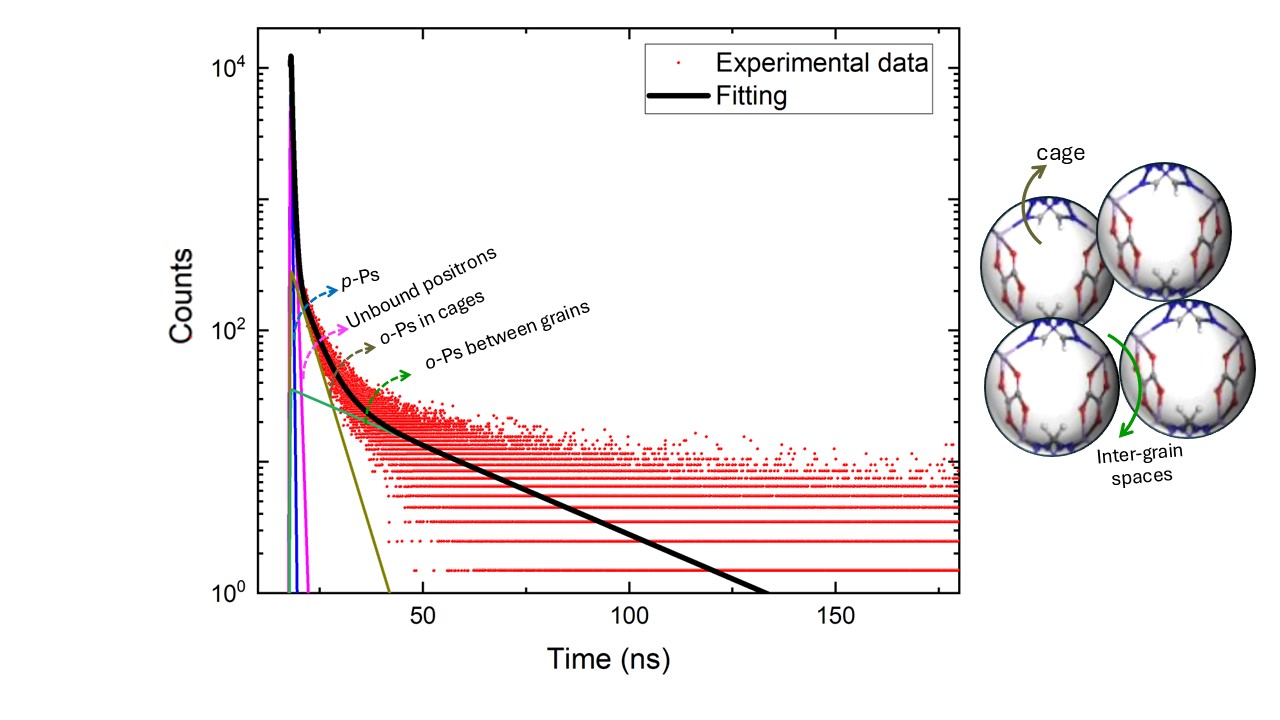


**Figure S 2**. Typical positron annihilation lifetime histogram including the origins of the resolved lifetime components (τ_1-4_) in CALF-20. The third and fourth lifetime components are described in the text as τ_cage_ and τ_grain_, respectively.

annihilation in intergranular spaces (possibly defects at grain boundaries). From the lifetime we can estimate that they are either spheres with a size of 2.1-2.6 nm, gaps with a size of 1-1.5 nm, or mesopores with more complicated shapes and sizes between the previous two. According to the annihilation models,^[3,6]^ the negligible dependence of this lifetime on temperature (Table **1**) suggests that the shape is more gap-like. The origin of this component from intergranular spaces is supported by its moderate increase (from 9.9 % at 253 K to 11.6 % at 373 K, Table **1**) in the intensity of this component with temperature in a vacuum. This reflects the increase in the kinetic energy of o-Ps and, in consequence, the increase in the probability of its migration through the cages outside the grains. At 373 K at 5 mbar in Fig. **S5** and Fig. **S6**, this probability becomes comparable to the probability of annihilation in cages (I_4_ ≈ I_3_).

No clear pressure dependence on the intergranular component’s lifetime can be found (Fig. **S3**). The most pronounced increase with CO_2_ pressure at 253 K is visible in Fig. **S3**, but it concerns the component with relatively small intensity (2-3% when cages are filled with CO_2_, see Fig. **S5**). This may be related to the coverage of grains with CO_2_ and screening metal nodes in CALF-20, which results in reducing electron density on the surface of grains. A small decrease in the lifetime is visible at each temperature for the threshold pressure of filling cages by CO_2_. This may indicate an increase in the grain size, but also a flattening of the openwork structure of the grain’s


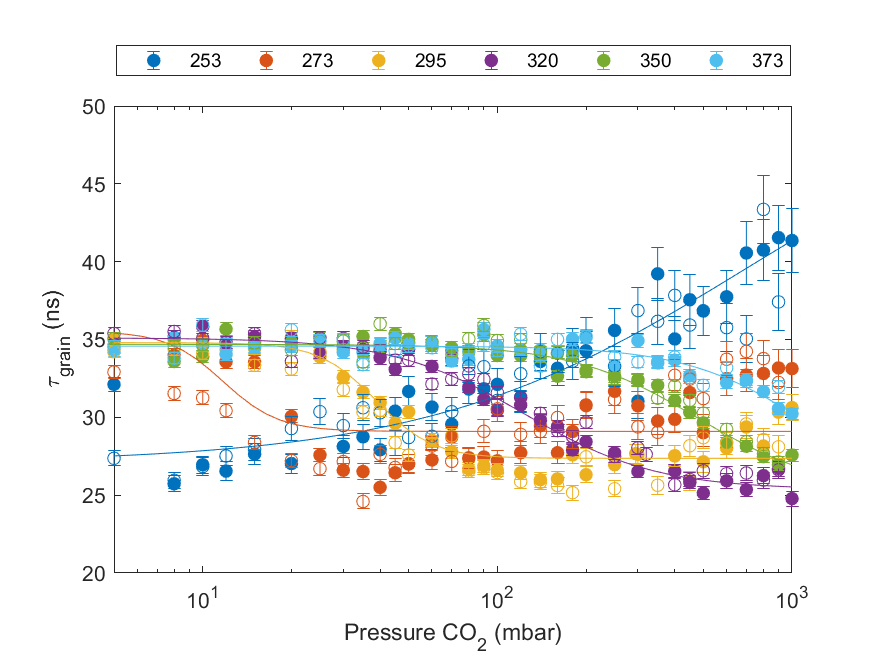


**Figure S 3**. Pressure dependence of the lifetime of ortho-positronium migrated to intergranular spaces of CALF-20 during adsorption (full symbols) and desorption (open symbols) at different temperatures. Sigmoidal functions fitted to adsorption data serve as eye-guides.

surface. However, this can be related in part to the significant decrease in the intensity of this component and distortion caused by the simplified model (single exponentials) fitted to the spectra. Additionally, the change in τ_grain_ can be related to the disappearance of Bloch states, which is described below. In this case, the observed increase in τ_grain_ at low temperatures at high pressures could indicate a disruption in the formation of o-Ps Bloch states at low temperatures, which leads to a drop in their contribution to the intergranular component. Only o-Ps migrated from the cage getting localized between grains and o-Ps formed at grain spaces survived, which have long lifetimes. The amount of these grain space-located o-Ps are not significant as reflected from their low intensity at T < RT at p_CO2_ > 100 mbar (I_grain_, Fig. **S5**).

***Main cages and Ps bubble***

The well-known bubble model,^[7]^ which is used to describe annihilation in liquids and confined liquids, can be modified for extremely narrow cages of CALF-20. This results in an equation for the minimum energy condition allowing to estimate the equilibrium condition for the gap length L:

| $\frac{\boldsymbol{\partial}}{\boldsymbol{\partial L}}\left( \boldsymbol{E}_{\boldsymbol{Ps}}\boldsymbol{+L}\boldsymbol{F}_{\boldsymbol{int}}\boldsymbol{+L}\boldsymbol{a}^{\boldsymbol{2}}\boldsymbol{p} \right)\boldsymbol{=}$0 | S1 |
| --- | --- |

where E_Ps_ is the energy of o-Ps zero motion, F_int_ is the force of CO_2_ interaction with cage walls, a is the side of the cage wall, and p is the pressure in CO_2_.

Above the sigmoidal transition the lifetime τ_cage_ between gaps decreases with pressure at 273 K from 2.10 ns at 60 mbar to 1.85 ns at 1000 mbar (Fig. **1a**). According to the bubble model (eq. **S1**) it would require about 1.4×10^5^ mbar (discussed in detail below) increase in pressure due to high o-Ps zero energy. Such behavior has been noted in the capillary condensation of some liquids when a negative pressure can occur in mesopores.^[8,9]^ However, given the large molecular mass of CO_2_, negative pressure within CALF-20 cages is not expected at all according to the Young-Laplace law (which is a macroscopic law, but successfully verified by PALS in the nanometer range). Moreover, capillary condensation is not expected in the microporous CALF-20 cages. Additionally, the observed gap size at 1000 mbar changes with increasing temperature significantly, what implies an unusually strong temperature dependence of the CO_2_-cage wall interaction. However, some justification for this may be the temperature change in the cage size without pressure. It suggests the change in the distance between the cage walls, which may be accompanied by the change in the positions of the oxalate and triazole groups most likely interacting with CO_2_ molecules.

Despite the previous doubts, we verify possible bubble formation. The hypothetical dependences of particular energy components and total energy minimum are presented in Fig. **S4**. In fact, the separation between the second and third terms in eq. **S1** (E_int_ and E_press_ in Fig. **S4**) is difficult, as they change in the same way with radius. Therefore, they both have a relative nature, e.g. pressure is assumed zero at an external pressure of 1000 mbar,


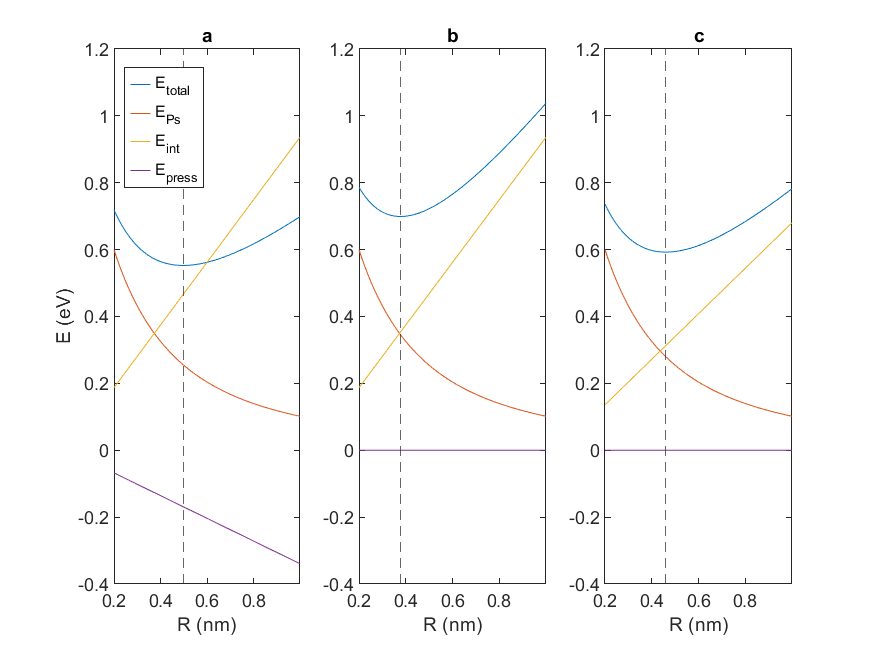


**Figure S 4**. Hypothetical energy of o-Ps zero motion (E_Ps_), CO_2_ interaction with cage walls (E_int_), and from internal pressure (E_press)_ used in the bubble model and sum of these energies (E_total_) as a function of gap length for (a) T = 253 K and p = 5 mbar, (b) T = 253 K and p = 1000 mbar, (c) T = 320 K and p = 1000 mbar. The vertical dashed line represents the minimum of E_total_.

which results in p = -137 MPa at 5 mbar to obtain the observed gap length. There is no reason to expect zero pressure at 1000 mbar, so the F_int_ of 0.150 N at 253 K and 0.109 N at 320 K, which determines the slope of the interaction term chosen to obtain experimental gap lengths, are certainly underestimated if the model is applicable. Nevertheless, their relative ratio should be preserved and the decrease in this interaction F_int_ estimated from the bubble model as large as 27% in the range of ca. 70 K is unlikely even taking into account the temperature change in the cage size. Therefore, it is not justified to ascribe the changes in the gap size to the interaction of CO_2_ with o-Ps.

***o-Ps intensities***

The changes in intensity of the intergranular component (*I_grain_*) in Fig. **S5** are very well correlated with changes in *τ_cage_* (Fig. **1a**).


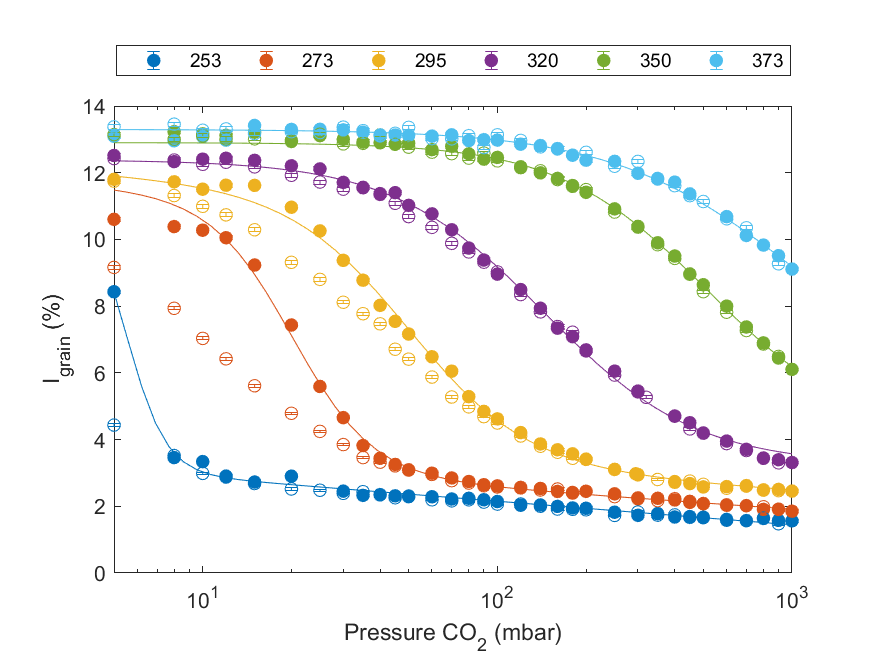


**Figure S 5**. Pressure dependence of the intensity of ortho-positronium migrated to intergranular spaces of CALF-20 during adsorption (full symbols) and desorption (open symbols) at different temperatures. Sigmoidal functions were fitted to adsorption data.

The intensity of the cage component (*I_cage_*) changes are shifted towards a few times greater pressures according to changes in *I_grain_* (Fig. **S6**). This shift demonstrates some interesting remarks about o-Ps formation. I_grain_ cannot reflect solely annihilation of o-Ps migrating from cages, because in such case we would expect mirror I_cage_ and I_grain_ dependences. Moreover, according to the scanning electron microscope images in Fig. **S10**, the maximum crystal size is ca. 5 µm. This means that one expects relatively low o-Ps migration from the cages to the intergranular spaces, and I_grain_ seems to be overestimated. In this case, other factors should be considered. In CALF-20, implanted positrons can either annihilate from the unbound state or survive until forming Ps states. Then, Ps formed between the chains of the bulky part of CALF-20 can either (i) loose energy to get trapped and localized in the cages, wherein less potential energy for Ps is expected, or (ii) reach the cage centers without interactions with atoms remaining hot Ps having sufficient energy to spread over the cages. This means that this fraction acts as delocalized Ps Bloch-like state. The Bloch state of Ps in MOFs and zeolites have been already discussed in other researches.^[10–12]^ This means that I_grain_ is an average of o-Ps migrating from the cages to intergranular spaces and delocalized Ps inside the cages.


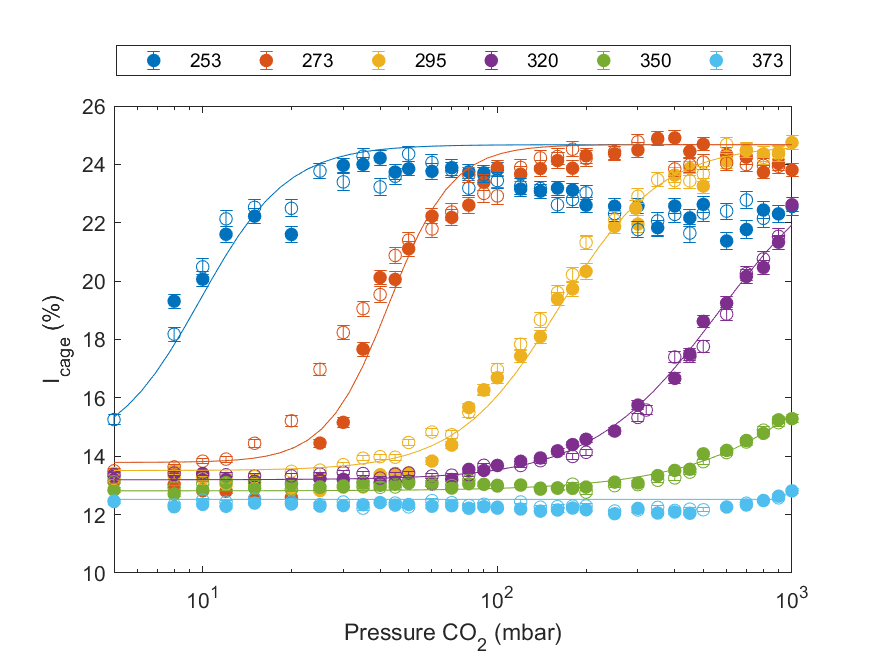


**Figure S 6**. Pressure dependence of the intensity of ortho-positronium trapped in cages of CALF-20 during adsorption (solid symbols) and desorption (open symbols) at different temperatures. Sigmoidal functions were fitted to adsorption data.

The equation **S2**, which was used to describe the sigmoidal function in the main text

| $\boldsymbol{I}_{\boldsymbol{cage}}\boldsymbol{=}\frac{\boldsymbol{I}_{\boldsymbol{empty}}\boldsymbol{-}\boldsymbol{I}_{\boldsymbol{filled}}}{\boldsymbol{1+(}{\frac{\boldsymbol{p}}{\boldsymbol{p}_{\boldsymbol{1/2}}}\boldsymbol{)}}^{\boldsymbol{k}}}\boldsymbol{+}\boldsymbol{I}_{\boldsymbol{filled}}$ | S2 |
| --- | --- |

can be rewritten in the following form;

| $\boldsymbol{I}_{\boldsymbol{cage}}\boldsymbol{=}\frac{\boldsymbol{I}_{\boldsymbol{empty}}\boldsymbol{-}\boldsymbol{I}_{\boldsymbol{filled}}\boldsymbol{+}\boldsymbol{I}_{\boldsymbol{filled}}\boldsymbol{(1+(}{\frac{\boldsymbol{p}}{\boldsymbol{p}_{\boldsymbol{1/2}}}\boldsymbol{)}}^{\boldsymbol{k}}\boldsymbol{)}}{\boldsymbol{1+(}{\frac{\boldsymbol{p}}{\boldsymbol{p}_{\boldsymbol{1/2}}}\boldsymbol{)}}^{\boldsymbol{k}}}\boldsymbol{.}$ | S3 |
| --- | --- |

If we set R =$( {\frac{1}{p_{1/2}})}^{k}$ , we will have

| $\boldsymbol{I}_{\boldsymbol{cage}}\boldsymbol{=}\frac{\boldsymbol{I}_{\boldsymbol{empty}}\boldsymbol{-}\boldsymbol{I}_{\boldsymbol{filled}}\boldsymbol{+}\boldsymbol{I}_{\boldsymbol{filled}}\boldsymbol{+}\boldsymbol{I}_{\boldsymbol{filled}}\boldsymbol{\times R\times}\boldsymbol{p}^{\boldsymbol{k}}}{\boldsymbol{1+R\times}\boldsymbol{p}^{\boldsymbol{k}}}$ | S4 |
| --- | --- |

and its final form will be:

| $\boldsymbol{I}_{\boldsymbol{cage}}\boldsymbol{=}\frac{\boldsymbol{I}_{\boldsymbol{empty}}\boldsymbol{+}\boldsymbol{I}_{\boldsymbol{filled}}\boldsymbol{\times R\times}\boldsymbol{p}^{\boldsymbol{k}}}{\boldsymbol{1+R\times}\boldsymbol{p}^{\boldsymbol{k}}}$ = $\frac{\boldsymbol{I}_{\boldsymbol{filled}}\boldsymbol{\times R\times}\boldsymbol{p}^{\boldsymbol{k}}}{\boldsymbol{1+R\times}\boldsymbol{p}^{\boldsymbol{k}}}\boldsymbol{+}$ $\frac{\boldsymbol{I}_{\boldsymbol{empty}}}{\boldsymbol{1+R\times}\boldsymbol{p}^{\boldsymbol{k}}}$ , | S5 |
| --- | --- |

which is eq. **3** in the main text.

Worth noting, the validity of the comparison of eq. **3** in the main text and the Langmuir–Freundlich isotherm (eq. **4)** is supported by the fact that *I_filled_* can qualitatively mirror the maximum adsorbable amount of CO_2_ (*q_m_*), and *C ∝ p*. In this case, *K_s_ ∝* $R$, where the proportionality constant should have the unit *mbar×L×mg^-1^*.

The total o-Ps intensity reflects the o-Ps formation probability, which, typically, correlates with the total surface of free volumes (both open and closed). It exhibits a decrease in the pressure range of the sigmoidal transition (Fig. **S7**). This decrease is quite significant, from an average of 25-26% to the minimum value of about 20-21%. Such a decrease in the total surface due to the cage filling by CO_2_ is obvious – the cage walls are blocked/covered with CO_2_ molecules. In this case, the amount of o-Ps formed on the pore walls (delocalized o-Ps) will be less. More interesting is the further restoration of the initial intensity, which is mainly driven by the increase in I_cage_ at higher pressures at low temperatures, except for 253 K. Such an increase in o-Ps intensities is observed each time pores are filled with different liquids in an amount that allows for bubble formation, even if their size is comparable with


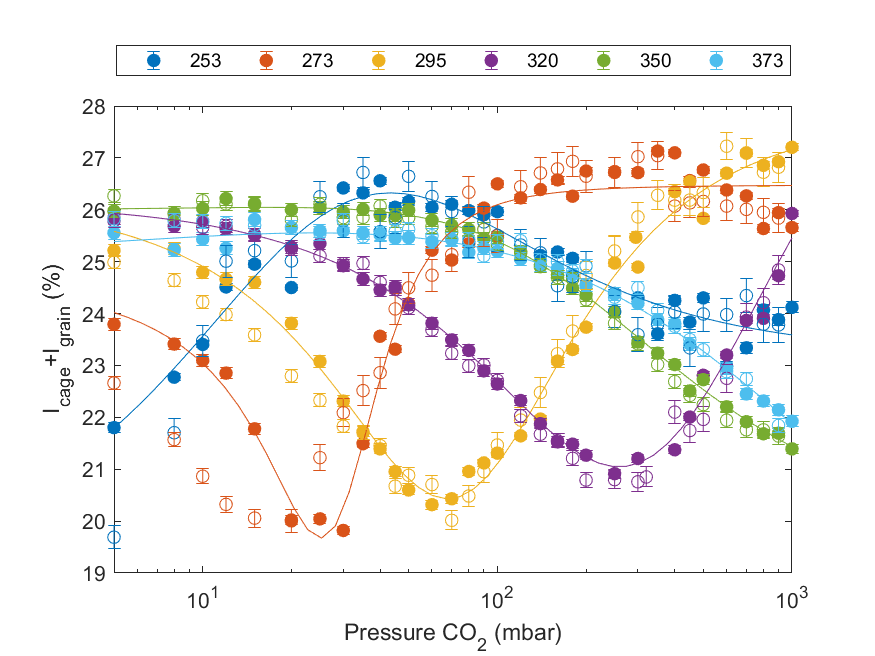


**Figure S 7**. Pressure dependence of the sum of intensities of both o-Ps components during adsorption (full symbols) and desorption (open symbols) at different temperatures. Lines are eye-guides.

CALF-20 cages.^[9,13]^ As discussed above, we do not expect bubble formation in CO_2_. Therefore, this can be a consequence of the reorganization of CO_2_ molecules, namely due to the appearance of a greater number of gaps. Additionally, some CO_2_ molecules can shift to the sides of the cages in CALF-20, as discussed in the main text, making the empty spaces on the cage surface denser and the Ps formation higher. In this case, o-Ps formation will be higher (the denser cage sides) and there will be more trapping sites to host o-Ps (gaps between CO_2_ molecules and inside isolated cages), therefore I_cage_ increases resulting in an increase in the total o-Ps intensity.

***Fitting the sigmoidal function***

The parameters resulting from fitting the sigmoidal dependence (equation **1**, main text) to the pressure dependence of *τ_cage_* and *I_grain_* are presented in Table **S1** and Table **S2**, respectively. The temperature dependence of the width of sigmoidal change *k* obtained from *τ_cage_* , *I_cage_*, and *I_grain_* pressure dependences are shown in Fig. **S8**.

**Table S 1**. Fitting parameters obtained for the pressure dependence of τ_cage_ at various temperatures: p_½_ is the CO_2_ pressure at the middle of the sigmoidal change, k is the width of sigmoidal change, τ_empty_ is a lifetime in cages without CO_2_, τ_filled_ is a lifetime in cages with CO_2_

| T  (K) | p_½_  (mbar) | Δp_½_  (mbar) | k | Δk | τ_empty_  (ns) | Δτ_empty_  (ns) | τ_filled_  (ns) | Δτ_filled_  (ns) |
| --- | --- | --- | --- | --- | --- | --- | --- | --- |
| 253 | 5.9 | 0.1 | 0.117 | 0.006 | 4.34 | 0.16 | 1.74 | 0.06 |
| 273 | 23.7 | 0.3 | 0.209 | 0.011 | 4.45 | 0.14 | 1.85 | 0.05 |
| 295 | 61.2 | 1.0 | 0.411 | 0.037 | 4.57 | 0.12 | 1.98 | 0.05 |
| 320 | 174.9 | 3.4 | 0.574 | 0.017 | 4.70 | 0.11 | 2.11 | 0.06 |
| 350 | 567.9 | 9.9 | 0.620 | 0.019 | 4.86 | 0.11 | 2.28^*^ | 0.08^*^ |
| 373 | 1235.5 | 66.9 | 0.624 | 0.047 | 4.98 | 0.12 | 2.41^*^ | 0.10^*^ |

*results of extrapolation presented in Fig. **S9**.

**Table S 2.** Fitting parameters obtained for the pressure dependence of I_grain_ at various temperatures: p_½_ is the CO_2_ pressure at the middle of the sigmoidal change, k is the width of sigmoidal change, I_empty_ is intensity in cages without CO_2_, I_filled_ is intensity in cages with CO_2_, and I_e-f_ is the difference between I_empty_ and I_filled_.

| T  (K) | p_½_  (mbar) | Δp_½_  (mbar) | k | Δk | I_empty_  (%) | ΔI_empty_  (%) | I_filled_  (%) | ΔI_filled_  (%) | I_e-f_  (%) | ΔI_e-f_  (%) |
| --- | --- | --- | --- | --- | --- | --- | --- | --- | --- | --- |
| 253 | 5.5 | 0.1 | 0.154 | 0.009 | 11.24 | 1.22 | 1.48 | 0.41 | 9.76 | 1.63 |
| 273 | 20.1 | 0.4 | 0.315 | 0.020 | 11.58 | 1.05 | 2.01 | 0.36 | 9.57 | 1.41 |
| 295 | 50.0 | 0.8 | 0.465 | 0.037 | 11.96 | 0.90 | 2.58 | 0.36 | 9.38 | 1.26 |
| 320 | 144.0 | 1.8 | 0.593 | 0.011 | 12.39 | 0.79 | 3.24 | 0.42 | 9.15 | 1.21 |
| 350 | 518.1 | 6.1 | 0.579 | 0.013 | 12.90 | 0.79 | 4.02^*^ | 0.54^*^ | 8.88^*^ | 1.33* |
| 373 | 1080.0 | 25.4 | 0.729 | 0.022 | 13.30 | 0.89 | 4.62^*^ | 0.66^*^ | 8.68^*^ | 1.55* |

*results of extrapolation presented in Fig. **S9**.

Despite overall similarity, a key difference between the change in *I_grain_* and *τ_cage_* is the change in the fitted extreme values of each parameter, i.e. without CO_2_ (*τ_empty_*, *I_empty_*) and with CO_2_ (*τ_filled_*, *I_filled_*). After CO_2_ fills cage, *I_grain_* decreases greatly by *I_e-f_* = *I_empty_* - *I_filled_* = 8.7-9.7% (i.e. to 0.13-0.34 of its value without gas *I_empty_*). The difference *I_e-f_* changes with temperature: the greatest one is observed at low temperatures, and then it linearly decreases with increasing temperature (Table **S2**). In contrast, the difference *τ_empty_* - *τ_filled_* is constant in the whole temperature range.


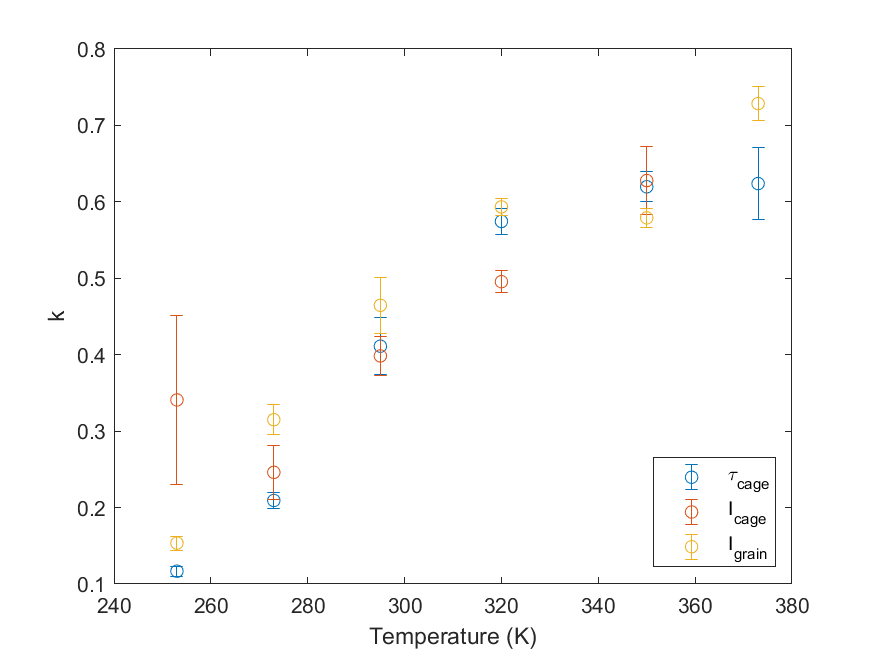


**Figure S 8**. Temperature dependence of the width of sigmoidal change observed in CALF-20 as a change in the o-Ps lifetime in cages (τ_cage_), intensity of the cage component (I_cage_) and the intergranular component (I_grain_). The value of k for I_grain_ at 373 K is omitted due to its great uncertainty.

To obtain a reliable fit for the sigmoidal changes that spread over 1000 mbar (i.e. at 350 and 373 K), the lifetimes in cages filled with CO_2_ (*τ_filled_*) as well as intensities of the intergranular component at the same CO_2_ pressure (I_filled_) were determined by the linear extrapolation of the temperature dependence of *τ_filled_* or I_filled_ observed at lower temperatures (Fig. **S9**).

Gap lengths (*L_gap_*) for chosen pressures were calculated from *τ_cage_* using the annihilation model with the assumption of a cuboid geometry^[14]^ (Table **S3**). For each temperature, a side of an infinite channel with a square cross-section was calculated^[3]^ from *τ_cage_* without CO_2_ and used as two sides of the cuboid to determine the third one, i.e. *L_gap_*.


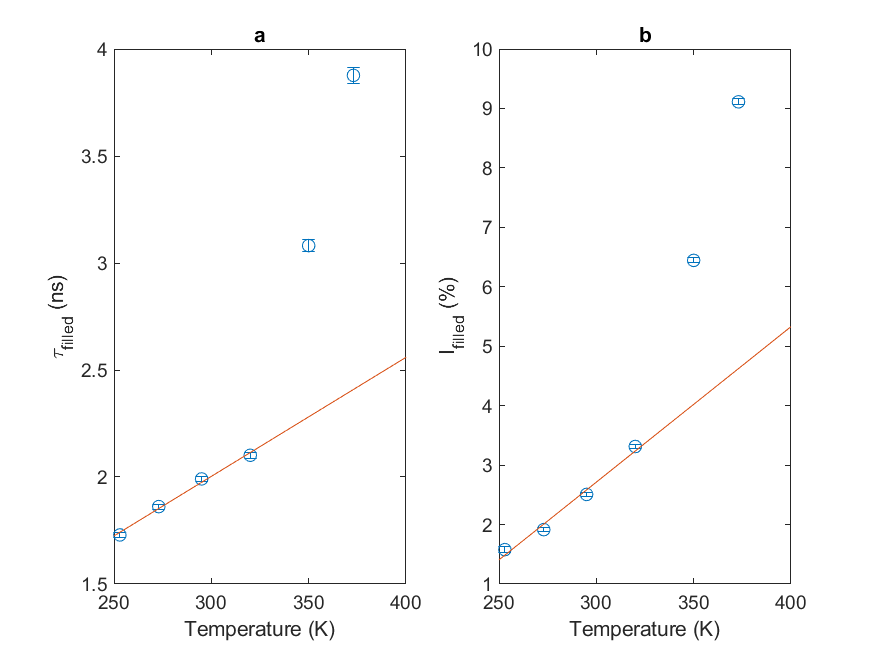


**Figure S 9**. Temperature dependence of (a) lifetimes in cages filled with CO_2_ and (b) corresponding intensities of the intergranular component. The linear function was fitted to the measured τ_filled_ and I_filled_ in the range of 253-320 K.

**Table S 3.** Gap lengths calculated from τ_cage_ for different temperatures at 1000 Pa and at pressure just above the sigmoidal change (when lifetime decreased by 99% of its total change).

| T (K) | L_gap_ (nm) | ΔL_gap_ (nm) | L_gap_ (nm) | ΔL_gap_ (nm) |
| --- | --- | --- | --- | --- |
|  | *1000 Pa* | | *99% reduction in τ_cage_* | |
| 253 | 0.379 | 0.008 | 0.490 | 0.012 |
| 273 | 0.404 | 0.006 | 0.470 | 0.016 |
| 295 | 0.430 | 0.006 | 0.453 | 0.021 |
| 320 | 0.459 | 0.006 | 0.466 | 0.023 |
| 350 | - | - | 0.500^*^ | 0.016^*^ |
| 373 | - | - | 0.526^*^ | 0.018^*^ |

*results of extrapolation presented in Fig. **S9**.

***Hysteresis in o-Ps components during CO_2_ desorption***

During desorption all o-Ps parameters exhibit hysteresis at *p* < 70 mbar at temperatures, starting from RT and below. Namely, *τ_cage_* (Fig. **1a**) and *I_grain_* (Fig. **S5**) exhibit pronounced and similar hysteresis suggesting that CO_2_ molecules remain within the cages at pressure below their adsorption pressure, while *I_cage_* displays narrower hysteresis between CO_2_ adsorption and desorption curves (Fig. **S6**). This observation suggests that physisorption is out of equilibrium, given that the time per step during PALS was 2 hours. Small hysteresis of *I_cage_* may point out that the CO_2_ molecules pushed out of the cage centers leave their positions as soon as there is no additional energy provided by pressure that can hold them in positions. This indicates that these locations of CO_2_ are unstable. Whereas significant hysteresis of *τ_cage_* and *I_grain_* indicate that some of CO_2_ molecules get "stuck" at the energetically favorable sites and act as “walls” for still small gaps between them as shown by small *τ_cage_*. They also suppress formation of the Bloch states as indicated by low *I_grain_*. The presence of such attractive interaction is confirmed by lack of hysteresis above room temperature, when thermal activation is sufficient to break it. Lifetime *τ_grain_* shows complex behavior (Fig. **S3**), reflecting the interplay between CO_2_ adsorption, desorption, and the heterogeneous grain structure. This complexity may arise from varying adsorption energies, and the balance between CO_2_ molecules and o-Ps in the MOF cages.

***Humidity and Humid CO_2_ in CALF-20***

In the pure humidity experiment, air at atmospheric pressure was used as a carrier. Typical atmospheric air contains about 400 ppm (0.04%) of CO_2_.^[15]^ Since we compare the pure humidity experiments with the humid CO_2_, it is important to justify that the low CO_2_ in air (during the so-called “pure humidity” experiment) makes the comparison still valid. The humid CO_2_ experiment was performed with 970 mbar CO_2_ pressure. At this pressure, the CO_2_ content is ca. 95.73 %. Therefore, the difference in CO_2_ content in both experiments was substantial i.e., 95.73 % / 0.04 % = 2393 times higher. This indicates that even if the pure humidity experiments contain CO_2_, its contribution can be considered as negligible.

Since the grain component is expected to include delocalized o-Ps annihilation, it is also possible that at high H_2_O pressure the annihilation of the delocalized o-Ps is influenced during its diffusion through and escaping from grains within CALF-20. If delocalized o-Ps reaches the grain spaces as a thermalized entity, due to collision with cages during diffusion, then its energy at grain spaces will be *E_0_ + kT*,^[16]^ with *E_0_* = 0.1 – 0.3 eV.^[17]^ With this energy, delocalized o-Ps can re-enter the cages, averaging the longest-lived o-Ps lifetime to the values observed for τ_grain_ in Fig. **S3** when there is no gas buffer. However, when gas or vapor exists inside the cages, the energy of o-Ps in the grain decrease to *kT* or below, hindering its re-entry. In this case, more (or all) escaped o-Ps tend to annihilate in intergranular spaces and thus increasing average positronium lifetime. This amount of escaped o-Ps is expected to be low due to the presence of gas within the cage.

**Appendix S 4. Calculation of the isosteric heat of CO_2_ adsorption in CALF-20**

The calculation of the isosteric heat of adsorption is calculated using Clausius-Clapeyron equation:^[18]^

${\Delta H}_{ads}= -R\cdot ln(\frac{p_{2}}{p_{1}})\frac{T_{1}\cdot T_{2}}{T_{2}-T_{1}}$ S6

where R = 8.314 J/molK, p_2_ – adsorption equilibration pressure at T_2_, p_1_ – adsorption equilibration pressure at T_1_.

Langmuir-Freundlich fit was conducted for CO_2_ physisorption isotherms measured at 253K, 273K, 298K, 320K, 350K and 373K (Fig. **S10**). The fit parameters are provided in the Table **S4**.

**Figure S 10**. Adsorption of CO_2_ on CALF-20 at different temperatures (black spheres) and corresponding Langrmuir-Freundlich fit (red line).

**Table S 4**. Fit parameters, derived from the Langmuir-Freundlich fit of the CO_2_ isotherms on CALF-20 at various temperatures.

| Temperature | a | b | c | R^2^ | χ^2^ |
| --- | --- | --- | --- | --- | --- |
| 253 K | 6.62083 ± 0.13207 | 0.82034 ± 0.04971 | 0.55564 ± 0.02987 | 0.99259 | 0.02423 |
| 273 K | 5.73005 ± 0.26128 | 0.34796 ± 0.02236 | 0.51671 ± 0.03432 | 0.98966 | 0.01668 |
| 298 K | 4.03111 ± 0.16449 | 0.24924 ± 0.0174 | 0.73181 ± 0.06009 | 0.98789 | 0.01466 |
| 320 K | 3.28651 ± 0.068 | 0.1074 ± 0.00922 | 0.93072 ± 0.04736 | 1.000 | 0.00352 |
| 350 K | 2.82564 ± 0.02017 | 0.02076 ± 7.71462E-4 | 1.17015 ± 0.01502 | 0.99972 | 1.45365E-4 |
| 373 K | 2.11242 ± 0.01989 | 0.00702 ± 2.32905E-4 | 1.23169 ± 0.01263 | 0.99984 | 3.60963E-5 |

We used the isotherms measured at 320 K and 350 K for the calculation of the isosteric heat because of the better fit convergence and smaller values of standard uncertainties. The resulted plot is given in Fig. **S11**. It shows a very high values of 60 kJ/mol at low loading with further exponential decrease to 37 kJ/mol at high CO_2_ loadings.

**Figure S 11**. Isosteric heat of CO_2_ adsorption on CALF-20, calculated from Clausius-Clapeyron equation.

**Appendix S** **5. Crystal morphology of CALF-20**

SEM images of CALF-20 crystals in Fig. **S12** show the broad distribution of the crystal size in the sample ranging from submicron crystals and reaching 5 µm in length.

**Appendix S** **6. Nitrogen (77K) and water vapor (298K) physisorption on CALF-20**

**Figure S 12**. SEM images of CALF-20 crystals.


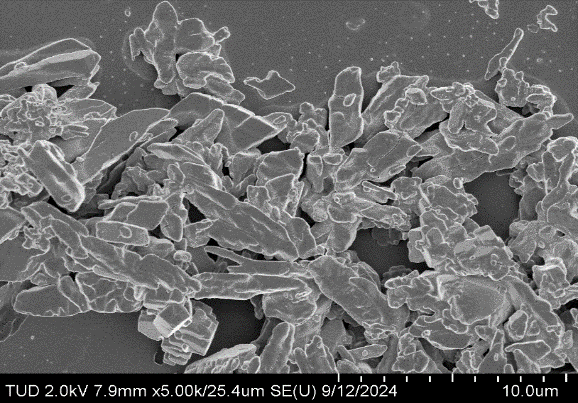

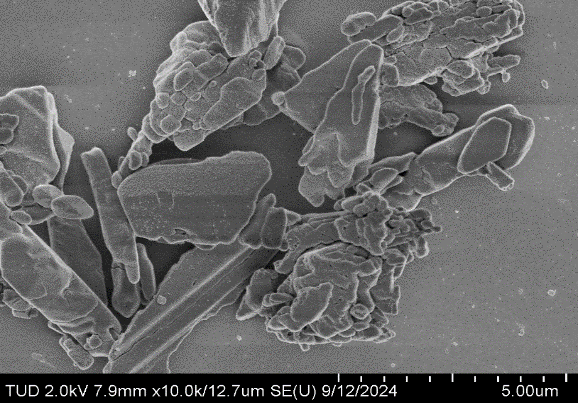

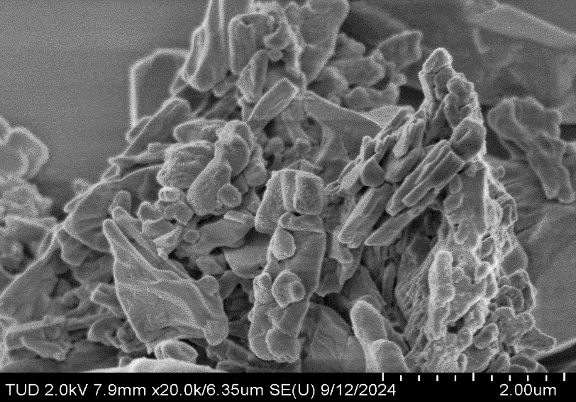

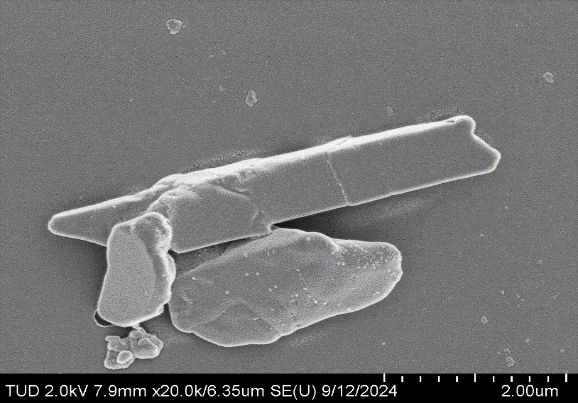


Nitrogen physisorption at 77 K was conducted in order to evaluate the porosity of the investigated CALF-20 sample (Fig. **S13**, top). The isotherm shows type Ia slope and reaches the saturation at p/p_0_ = 0.003 showing uptake of 5.2 mmol g^-1^, corresponding to the pore volume of 0.183 cm^3^ g^-1^. Water vapor physisorption at 298 K was conducted in order to confirm the chemical capacity and hydrophobic nature of the particular CALF-20 sample (Fig. **S13**, bottom). The obtained isotherm confirms the steep step at p/p_0_ = 0.1-0.15, followed by less steep water uptake, saturated at p/p_0_ = 0.9 with the uptake of 9.5 mmolg^-1^. A narrow hysteresis between adsorption and desorption branch of the isotherm can be explained by the slow kinetics of adsorption and desorption of the water in the micropores of CALF-20.

**Figure S 13**. Nitrogen at 77 K (top) and water vapor at 298 K (bottom) physisorption isotherms on CALF-20.

**Appendix S** **7. Powder XRD of CALF-20 at different temperatures and atmospheres**

Variable temperature PXRD on CALF-20 in the controlled gas atmospheres were conducted with the purpose to understand the phase behaviour of CALF-20 in the temperature range 293-404 K under inert gas conditions and exposed to dry and humid CO_2_ gas. Patterns are depicted in Fig. **S14**.


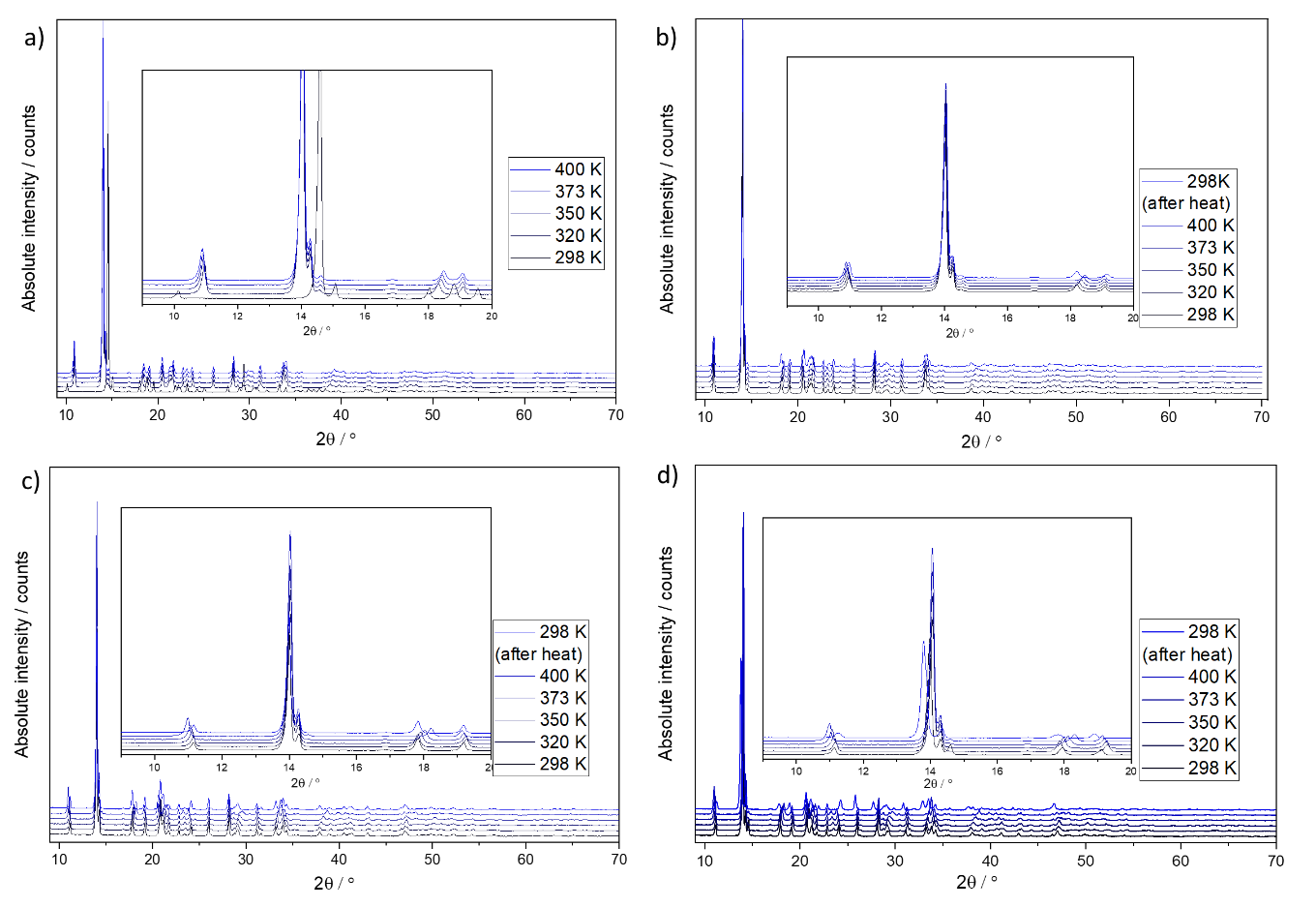


**Figure S 14**. Variable temperature PXRD patterns measured on CALF-20 in a) dry nitrogen flow; b) wet nitrogen flow; c) dry CO_2_ flow; d) wet CO_2_ flow.

References

[1] O. E. Mogensen, *J. Chem. Phys.* **1974**, *60*, 998.

[2] A. G. Attallah, V. Bon, K. Maity, R. Zaleski, E. Hirschmann, S. Kaskel, A. Wagner, *J. Phys. Chem. Lett.* **2024**, *15*, 4560.

[3] T. L. Dull, W. E. Frieze, D. W. Gidley, J. N. Sun, A. F. Yee, *J. Phys. Chem. B* **2001**, *105*, 4657.

[4] S. J. Tao, *J. Chem. Phys.* **1972**, *56*, 5499.

[5] M. Eldrup, D. Lightbody, J. N. Sherwood, *Chem. Phys.* **1981**, *63*, 51.

[6] T. Goworek, K. Ciesielski, B. Jasińska, J. Wawryszczuk, *Chem. Phys.* **1998**, *230*, 305.

[7] R. A. Ferrell, *Phys. Rev.* **1957**, *108*, 167.

[8] R. Zaleski, M. Gorgol, A. Kierys, P. Maheshwari, M. Pietrow, P. K. Pujari, B. Ena, Z. Zgardzińska, B. Zgardzińska, *J. Phys. Chem. C* **2022**, *126*, 5916.

[9] R. Zaleski, A. Kierys, M. Pietrow, B. Zgardzińska, A. Błażewicz, *J. Colloid Interface Sci.* **2020**, *558*, 259.

[10] R. Warringham, L. Gerchow, D. Cooke, P. Crivelli, R. S. Vallery, S. Mitchell, J. Pérezpérez-Ramírez, **2018**, DOI 10.1021/acs.jpcc.7b11336.

[11] P. Crivelli, D. Cooke, B. Barbiellini, B. L. Brown, J. I. Feldblyum, P. Guo, D. W. Gidley, L. Gerchow, A. J. Matzger, *Phys. Rev. B - Condens. Matter Mater. Phys.* **2014**, *89*, 241103.

[12] A. Zubiaga, R. Warringham, S. Mitchell, L. Gerchow, D. Cooke, P. Crivelli, J. Pérez-Ramírez, *ChemPhysChem* **2017**, *18*, 470.

[13] P. Maheshwari, M. Gorgol, A. Kierys, R. Zaleski, *J. Phys. Chem. C* **2017**, *121*, 17251.

[14] B. Jasinska, A. E. Koziol, T. Goworek, *J. Radioanal. Nucl. Chem.* **1996**, *210*, 617.

[15] J. Hou, Y. Sun, X. Dai, J. Liu, X. Shen, H. Tan, H. Yin, K. Huang, Y. Gao, D. Lai, W. Hong, X. Zhai, D. Norbäck, Q. Chen, *Indoor Air* **2021**, *31*, 1018.

[16] D. Dutta, J. I. Feldblyum, D. W. Gidley, J. Imirzian, M. Liu, A. J. Matzger, R. S. Vallery, A. G. Wong-Foy, *Phys. Rev. Lett.* **2013**, *110*, DOI 10.1103/PHYSREVLETT.110.197403.

[17] S. Mariazzi, P. Bettotti, R. S. Brusa, *Phys. Rev. Lett.* **2010**, *104*, 243401.

[18] S. Builes, S. I. Sandler, R. Xiong, *Langmuir* **2013**, *29*, 10416.
